# Living on the edge: light-harvesting efficiency and photoprotection in the core of green sulfur bacteria†

**DOI:** 10.1039/d3cp01321a

**Published:** 2023-06-26

**Authors:** Alexander Klinger, Dominik Lindorfer, Frank Müh, Thomas Renger

**Affiliations:** a Institut für Theoretische Physik, Johannes Kepler Universität Linz Altenberger Str. 69 4040 Linz Austria thomas.renger@jku.at

## Abstract

Photosynthetic green sulfur bacteria are able to survive under extreme low light conditions. Nevertheless, the light-harvesting efficiencies reported so far, in particular for Fenna–Matthews–Olson (FMO) protein-reaction center complex (RCC) supercomplexes, are much lower than for photosystems of other species. Here, we approach this problem with a structure-based theory. Compelling evidence for a light-harvesting efficiency around 95% is presented for native (anaerobic) conditions that can drop down to 47% when the FMO protein is switched into a photoprotective mode in the presence of molecular oxygen. Light-harvesting bottlenecks are found between the FMO protein and the RCC, and the antenna of the RCC and its reaction center (RC) with forward energy transfer time constants of 39 ps and 23 ps, respectively. The latter time constant removes an ambiguity in the interpretation of time-resolved spectra of RCC probing primary charge transfer and provides strong evidence for a transfer-to-the trap limited kinetics of excited states. Different factors influencing the light-harvesting efficiency are investigated. A fast primary electron transfer in the RC is found to be more important for a high efficiency than the site energy funnel in the FMO protein, quantum effects of nuclear motion, or variations in the mutual orientation between the FMO protein and the RCC.

## Abstract 2

Minimal model of light harvesting and photoprotection in the core of green sulfur bacteria.

## Graphical abstract

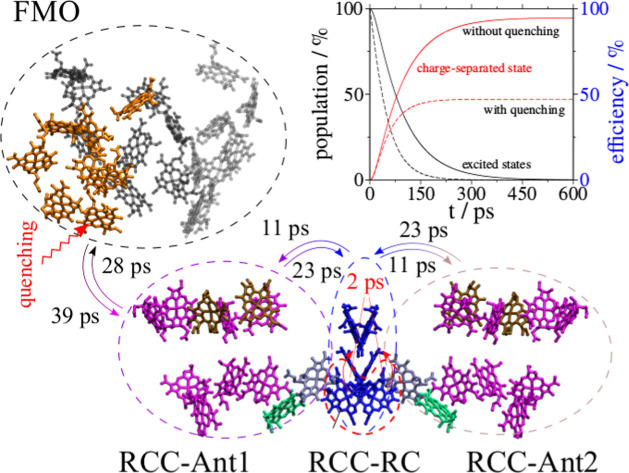

## 1 Introduction

Photosynthetic light-harvesting antennae enlarge the absorption cross-section of the reaction center (RC) providing excitation energy to drive the primary electron transfer reactions.^1^ A large variety of light-harvesting complexes have developed depending on the specific environment.^2,3^ Green sulfur bacteria (GSB) have succeeded to conquer regions with very low light intensities as they are found 100 m below the surface of the black sea^4,5^ or in the neighborhood of black smokers in 2000 m depth in the ocean.^6^ Living in an environment with very few photons available, a large cross-section of the reaction center is required, that is, a large antennae system and an efficient energy transfer mechanism. GSB have mastered this challenge by self-organizing their major light-harvesting pigments, bacteriochlorophylls (BChls) *c* and *e*, in lamella/rod like tubes that are stapled in a bag made of proteins.^9–17^ These bags are termed chlorosomes and contain 200 000–250 000 BChl *c* (BChl *e*) pigments.^10,15^ About 1% of the BChls are BChl *a* that are located in the periphery of the bag termed baseplate.^10,18,19^ Since BChl *a* has a lower excitation energy than BChls *c* and *e*, a funnel for excitation energy transfer towards the RC, located in the photosynthetic membrane, is created.

The water-soluble FMO protein is placed between the baseplate and the membrane containing the reaction center complex (RCC), creating space that allows electron carriers to reach the reaction center. The FMO protein, which is a trimer containing 8 BChl *a* pigments per monomer,^20^ acts as an excitation energy wire between the chlorosomes/baseplate and the RCC. A site energy funnel in the FMO protein is created by electrostatic pigment–protein interactions.^21,22^ The pigments that are closer to the RCC have a lower local transition energy (site energy) than those facing the baseplate/chlorosomes.^21–26^

So far, there is no consensus in the literature how efficient the light-harvesting process in GSB is. Estimates^27–31^ range from 20%,^27^ determined on isolated FMO-RCC supercomplexes, up to 75%^30^ measured on whole cells. Interestingly, some of the efficiencies measured on whole cells are larger than those measured on membrane fragments and FMO-RCC supercomplexes, despite the fact that the latter two do not contain the main light-harvesting system – the chlorosomes. This fact seems to suggest that the preparations of membrane fragments and FMO-RCC supercomplexes may lead to disruption of energy transfer pathways.

**Fig. 1:**
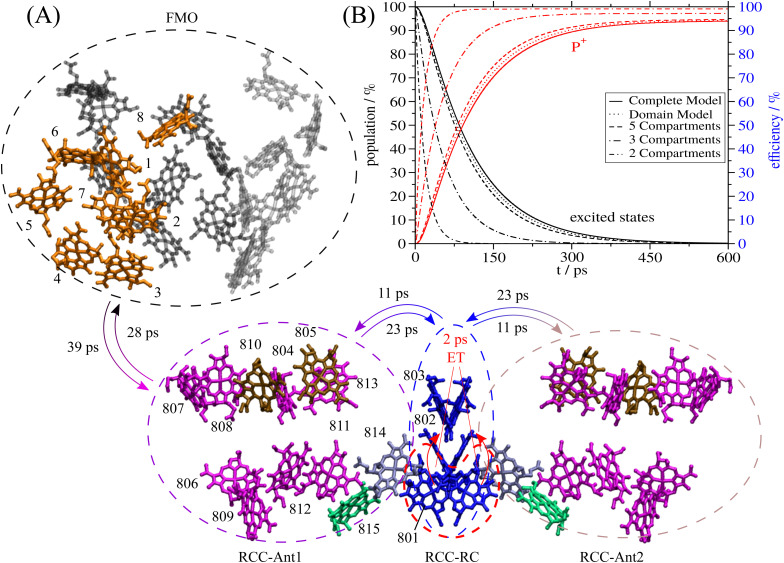
Structure of the FMO-RCC supercomplex and light harvesting. (A) Arrangement of BChl *a* (all but 802 and 803) and Chl *a* (802 and 803) pigments in the cryo-EM structural model^7^ of the FMO-RCC supercomplex containing the trimeric FMO protein and the dimeric RCC. The numbering of the pigments in the RCC is according to the protein data bank file 6M32 and that in the FMO protein follows the usual convention in the literature. Pigments that are colored equally are assigned to the same exciton domain in the calculation of optical spectra and energy transfer as explained in the main text. Numbers on the arrows are energy transfer time constants obtained in the 5-compartment model. The colored dashed lines depict the 4 excited states compartments denoted as FMO (black), RCC-Ant1 (purple), RCC-Ant2 (brown) and RCC-RC (blue). The 5th compartment contains the first charge-separated state formed by electron transfer from the special pair, which is encircled by a red-dashed line. Graphics of the molecules were made using VMD.^8^ (B) Sum of exciton state populations (black lines) and population of the charge separated state (red lines) obtained in different models as a function of time, assuming initial population of the FMO protein by transfer from the external baseplate, as described in the text.

On the other hand, a recent cryo-electron microscopy (cryo-EM) study^7^ of the FMO-RCC supercomplex of *Chlorobaculum tepidum*, which for the first time provided a high-resolution structure, found relatively large interpigment distances between the homotrimeric FMO and the homodimeric RCC (Fig. 1). The latter contains the reaction center (RC) and two core light-harvesting complexes (RCC-Ant1 and RCC-Ant2). The structure of the FMO-RCC supercomplex includes 8 BChl *a* pigments per monomer in the FMO trimer, 12 BChl *a* pigments in each antenna, and 2 BChl *a* and 4 Chl *a* pigments in the RC. Please note, that in GSB a second FMO trimer is bound to the RCC on top of RCC-Ant2 in Fig. 1. As has been noted in the cryo-EM study,^7^ this second FMO trimer probably got lost during the sample preparation due to the weak association between the FMO protein and the RCC. Evidence for the presence of a second FMO trimer in the supercomplex has been reported in an earlier cryo-EM study with nanometer resolution.^32^ A very recent high-resolution cryo-EM study indeed found a second FMO trimer, bound asymmetrically with respect to the first FMO trimer to the RCC.^33^ The authors argue that the second FMO trimer is incorporated with higher flexibility than the first one.

The smallest interpigment center-to-center distances between FMO and RCC occur between the low-energy BChls *a* 3 and 4 in the FMO protein and BChls *a* 807, 808 and 810 in the RCC-Ant1 subunit, ranging from 30 Å to 34 Å (Fig. 1). The authors of the cryo-EM study^7^ argue that these large distances could be responsible for the low-energy transfer efficiency, determined experimentally, but suggest to perform structure-based calculations to investigate this question quantitatively. Such calculations are presented in this work. As will be shown, the light-harvesting efficiency of the cryo-EM structure^7^ is about 95%.

Concerning microscopic mechanisms contributing to the efficiency of photosynthetic light harvesting, 2D photon-echo experiments on the FMO protein^34^ triggered a discussion about the role of quantum effects.^35–38^ There is evidence that the coherences, detected in the 2D experiments on the FMO protein, represent vibrational coherences of the electronic ground state not involved in energy transfer.^38^ One of the most direct experimental proofs was provided by Scholes, Blankenship and co-workers,^39^ who investigated the long-lived coherences on the wildtype and mutants of the FMO protein, where the excitation energy of BChl 3, that dominates the lowest-energy exciton state, was changed. Identical oscillation patterns where found for the wildtype and the mutant, providing clear evidence that these oscillations are not connected to the excited electronic states and, hence, are not connected to energy transfer. A recent review on quantum biology^38^ comes to the conclusion that quantum effects of nuclear motion are important for the relaxation of excitons in the domains of strongly coupled pigments as formed, *e.g.*, in the monomeric subunits of the FMO protein. Using a classical description of nuclear motion within Redfield theory, which neglects nuclear reorganization effects during exciton relaxation, resulted in an equal population of all exciton states. Very similar behavior was obtained by using a semiclassical modified Redfield theory that takes into account nuclear reorganization effects.^40^ In contrast, a quantum description of nuclear motion in either theory leads to a Boltzmann distribution giving rise to a preferential population of the low-energy exciton states in the FMO protein, which are localized close to the RCC. Here, we will investigate how the site energy funnel and a classical description of nuclear motion in the FMO protein affect the light-harvesting efficiency. The pigments in different FMO monomers are weakly coupled. Therefore, intermonomer energy transfer in the FMO trimer can be described by generalized Förster theory,^41–45^ that uses perturbation theory for the intermonomer excitonic couplings and takes into account intramonomer nuclear reorganization effects. In this case, a classical description of nuclei was found to strictly obey the principle of detailed balance of the rate constants giving rise to a preferential “down-hill” transfer of excitation energy.^45^

Under certain conditions, GSB need to protect the RC from receiving excess excitation energy.^29,46,47^ Otherwise, the RC could be damaged by oxidative side reactions involving the iron–sulfur (Fe–S) clusters that act as acceptors of excited electrons.^47^ Using stationary and time-resolved fluorescence spectroscopy on the wildtype and mutants, where Cys353 in the vicinity of BChl 3 and Cys49 near BChl 2 were exchanged, strong evidence was presented for the involvement of these residues in the shortening of the fluorescence life time of the FMO protein from 2 ns under anaerobic conditions to 60 ps under oxidative stress.^47^ From the comparison of the fluorescence of the FMO protein and the phosphorescence spectrum of isolated BChl *a*, a rough estimate of the energy of triplet states of the BChl pigments in the FMO protein has been obtained.^48^ It was argued that this energy could be too low for reaction with triplet oxygen yielding the harmful singlet oxygen. Hence, the purpose of the Cys-related quenching of the excited states in the FMO protein might be solely or at least partly the protection of the RC. The present calculations will quantify this effect.

Time-resolved experiments on isolated RCC revealed an overall decay of exciton states with a 25–35 ps time constant.^49,50^ Two phenomenological models to interpret this decay are the transfer-to-the trap limited model and the trap-limited model. Whereas in the former the excitation energy transfer to the RC is slow compared to primary electron transfer, the latter model assumes opposite behavior. The relatively large interpigment distances between the RC and the antenna subunits of the RCC seem to suggest that energy transfer to the RCC is slow. The present structure-based calculations will provide compelling evidence for this suggestion.

The remaining of this paper is organized in the following way: we start by introducing the Frenkel exciton Hamiltonian used to describe excitation energy transfer and optical spectra. In a next step, the parameters of this Hamiltonian are obtained based on the molecular structure,^7^ a calculation of circular and linear dichroism and linear absorption spectra, and comparison with experimental data.^24,28,51^ Finally, the Hamiltonian is used to study light-harvesting and the trapping of excitation energy by primary electron transfer reactions in the RC, revealing the light-harvesting efficiency. We will investigate aerobic and anaerobic conditions and different factors that influence the light-harvesting efficiency.

## 2 Theory

### 2.1 Frenkel exciton Hamiltonian and energy transfer

The pigment–protein complex (PPC) is described by the following standard Frenkel exciton Hamiltonian^52,53^1
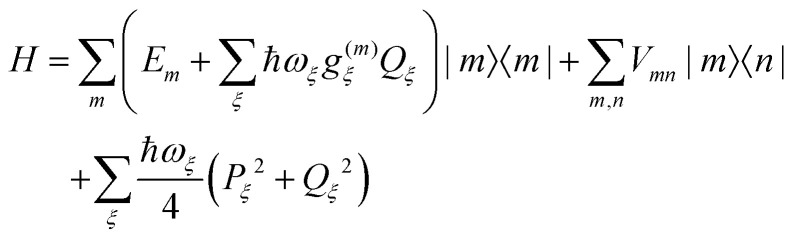
where |*m*〉 denotes an excited state of the PPC, which is localized at the *m*th pigment, that is, *m* is in the electronic excited state, whereas all other pigments *n* ≠ *m* are in their electronic ground state. The local optical transition energy of pigment *m* is assumed to vary around a mean value *E*_*m*_, where the fast fluctuations are described by a linear coupling to vibrational degrees of freedom *Q*_*ξ*_ with coupling constants *g*^(*m*)^_*ξ*_. The fluctuations that are slow on the time scale of the excited state lifetime (fs to ns) are treated as static disorder by randomly assigning local transition energies *E*_*m*_, taken from a Gaussian distribution function, which is centered around the mean transition energy *Ē*_*m*_. For each set of randomly assigned transition energies, the optical spectra and energy transfer kinetics are calculated and finally averaged. Correlations in static and dynamic transition energy fluctuations are neglected, since these effects were found to be negligibly small.^54,55^ The off-diagonal elements *V*_*mn*_ in the above Hamiltonian describe the coupling between excited states of the PPC that are localized at different sites *m* and *n*. These excitonic couplings *V*_*mn*_ are responsible for the transfer of excitation energy and for the delocalization of excited states. The last part of the Hamiltonian in eqn (1) contains the nuclear degrees of freedom, that are described by independent harmonic oscillators in the spirit of a normal mode analysis of the PPC^45,54^ with normal mode frequencies *ω*_*ξ*_ and dimensionless coordinates *Q*_*ξ*_ and momenta *P*_*ξ*_.^53^

The delocalization of excited states depends on the relative strength of excitonic coupling and dynamic and static disorder. A measure for the dynamic disorder is the local reorganization energy2
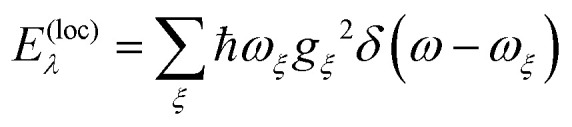
of the optical transition of pigments, where the square of the exciton–vibrational coupling constant *g*_*ξ*_ of the Hamiltonian enters, that is assumed to be equal for all sites *m*. If nuclei after the optical excitation of a pigment relax in potential energy surfaces of localized excited states, the PPC can lower its free energy by *E*^(loc)^_*λ*_. If, on the other hand, the excited states are delocalized, the excited state energy of the PPC decreases by an amount that is in the order of the nearest neighbor excitonic coupling *V*_*mn*_ between the pigments. Therefore, in the case of strong exciton–vibrational coupling, that is *E*^(loc)^_*λ*_ ≫ |*V*_*mn*_|, the excited states of the PPC will be localized. An explicit description of such a dynamic localization of exciton states would require a non-perturbative treatment of the excitonic and exciton–vibrational coupling, which is numerically costly.^56^ Here, we will implicitly take into account these effects by introducing exciton domains of strongly coupled pigments and allowing exciton delocalization only within these domains.^41^ A pigment belongs to an exciton domain, if it is excitonically coupled to at least one other pigment of that domain by an excitonic coupling *V*_*mn*_ that is larger than a certain cut-off value *V*_c_ chosen to be in the range of the local reorganization energy *E*^(loc)^_*λ*_. For the present spectral density (ESI,† Section S1.3.4) a reorganization energy of 32 cm^−1^ results, which has been assumed as the cut-off value *V*_c_ for the definition of exciton domains in the RCC (Fig. 1). In the case of the FMO trimer, we made an exception by including BChl 8 to the respective nearest monomer, despite the fact that its strongest coupling (21 cm^−1^ to BChl 1) is somewhat smaller than *E*^(loc)^_*λ*_. Note, however, that all intermonomer couplings are significantly weaker.

Exciton states 
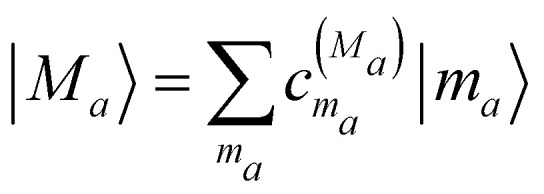
 are defined in domain *a* as the eigenstates of the exciton Hamiltonian 
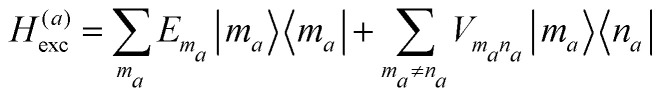
 that contains in the diagonal the site energies *E*_*m*_*a*__ and in the off-diagonal the excitonic couplings *V*_*m*_*a*_*n*_*a*__. The coefficient 
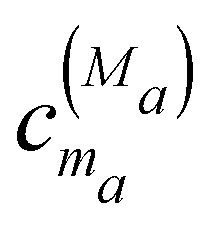
 is the *m*_*a*_th component of the *M*_*a*_th eigenvector of this exciton matrix, where *E*_*M*_*a*__ = *ħω*_*M*_*a*__ is the respective eigenvalue. Expanding the Hamiltonian in eqn (1) with respect to exciton states |*M*_*a*_〉 gives3
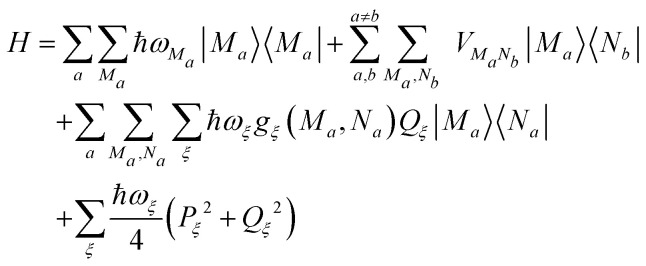
with the exciton–vibrational coupling constants in the basis of exciton states of domain *a*4
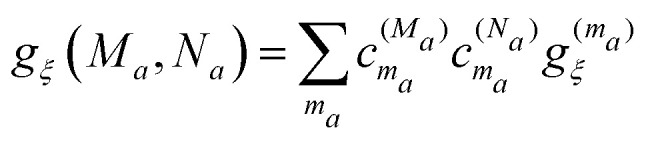
and the interdomain excitonic coupling5
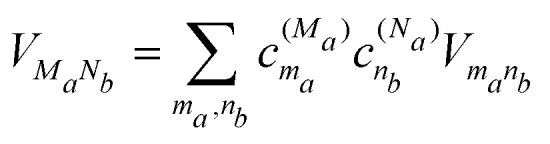
between exciton states |*M*_*a*_〉 in domain *a* and |*N*_*b*_〉 in domain *b*, where *V*_*m*_*a*_*n*_*b*__ is the individual excitonic coupling between pigments *m*_*a*_ and *n*_*b*_ in the two domains.

A normal mode analysis of the spectral density of the FMO protein has shown that the diagonal elements *g*_*ξ*_(*M*_*a*_,*M*_*a*_) are large compared to the off-diagonal elements *g*_*ξ*_(*M*_*a*_,*N*_*a*_).^54^ This inequality justifies a Markov and a secular approximation for the latter and an exact treatment of the former,^57^ giving rise to the Redfield type rate constant 
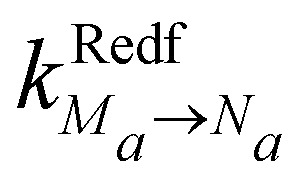
for exciton relaxation between exciton states |*M*_*a*_〉 and |*N*_*a*_〉 in domain *a* and the lineshape function *D*_*M*_*a*__(*ω*) of optical excitation of exciton states |*M*_*a*_〉 and 
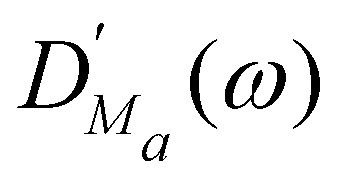
 of fluorescence from this exciton state. Explicit expressions for 
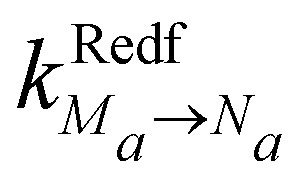
, *D*_*M*_*a*__(*ω*) and 
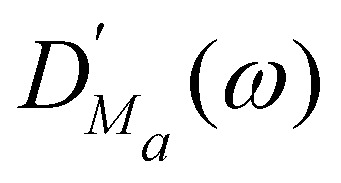
 are given in the ESI, eqn (S1)-(S11).†

The excitation energy transfer between exciton states |*M*_*a*_〉 and |*N*_*b*_〉 in different domains *a* and *b* is described by generalized Förster theory^41–45^ using second-order perturbation theory in the interdomain excitonic coupling *V*_*M*_*a*_*N*_*b*__ (eqn (5)) and treating the intradomain exciton–vibrational coupling as described above. Excellent agreement between this theory and a non-perturbative path-integral approach has been reported recently^58^ for the LH2 light-harvesting complex of purple bacteria. The interdomain rate constant 
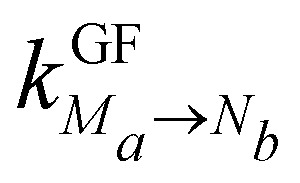
 is given as^41,45^6
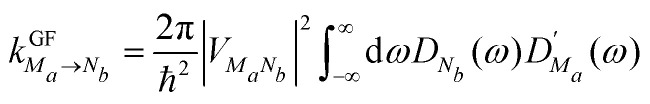
with the normalized intradomain lineshape functions for fluorescence of exciton state |*M*_*a*_〉 and absorption of |*N*_*b*_〉, discussed above. A semiclassical variant of this rate constant, in which the nuclear motion is treated classically,^45^ is given in the ESI,† eqn (S12).

We use a master equation approach^41^ to describe the exciton population dynamics in the FMO-RCC supercomplex. In addition to the Redfield and generalized Förster rate constants, the equations include rate constants for fluorescence, electron transfer in the RC and quenching of excitation energy at BChl 2 and 3 in the FMO protein. The latter process only occurs in the presence of molecular oxygen and protects the reaction center,^29,47^ as discussed in the introduction. The master equation for the population 
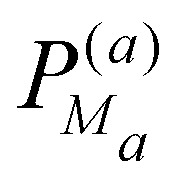
 of the *M*th exciton state in domain *a* reads7
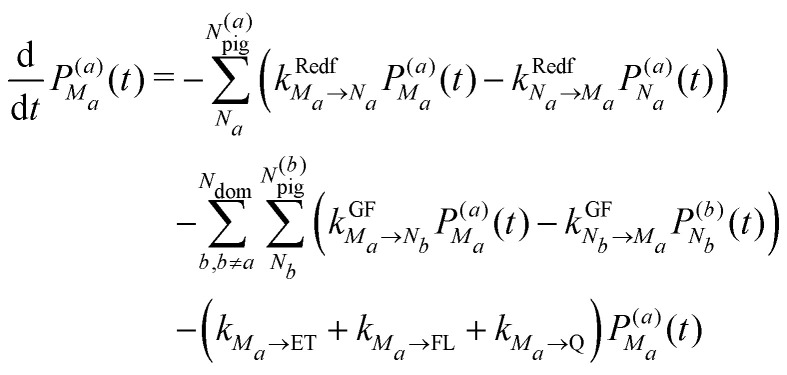
where *N*_dom_ is the number of domains, *N*^(*a*)^_pig_ and *N*^(*b*)^_pig_ are the number of pigments in domains *a* and *b* respectively, and 
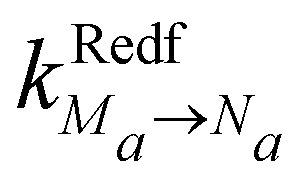
 (ESI,† eqn (S1)) and 
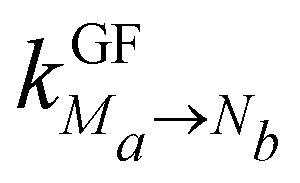
 (eqn (6)) are the intradomain and interdomain rate constants of excitation energy transfer, respectively. The rate constants *k*_*M*_*a*_→ET_, *k*_*M*_*a*_→FL_ and *k*_*M*_*a*_→Q_ describe primary electron transfer, fluorescence and quenching, respectively, from exciton state |*M*_*a*_〉 and are given as8
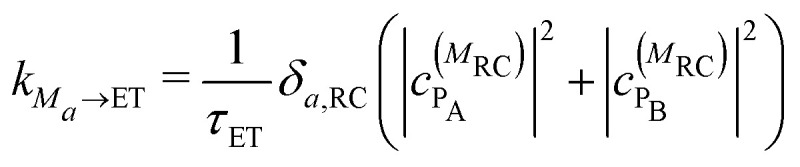
9
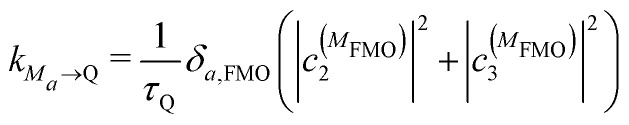
10
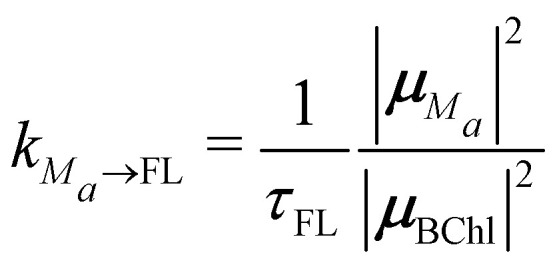
where 
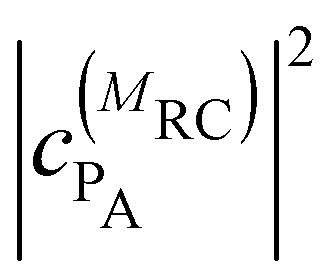
 and 
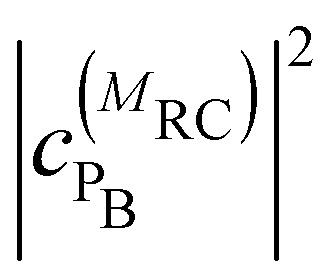
 are the probabilities to find the primary electron donors P_A_ and P_B_ in the central special pair formed by BChls *a* 801 of the two branches of the RC (Fig. 1) excited in state |*M*_RC_〉. 
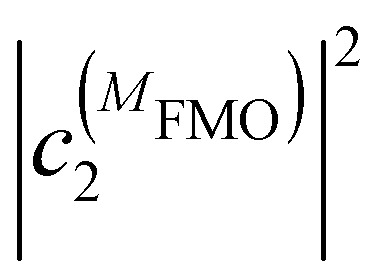
 and 
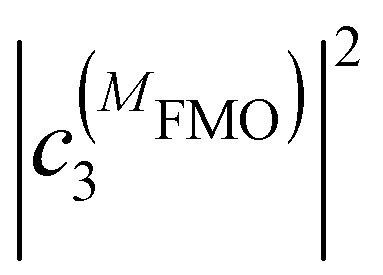
 are the probabilities that BChl *a* 2 and 3, respectively, in the FMO protein are excited in exciton state |*M*_FMO_〉. |***μ***_BChl_|^2^ is the dipole strength of an isolated BChl *a* pigment and |*μ*_*M*_*a*__|^2^ is that of exciton state |*M*_*a*_〉. *τ*_ET_ is the intrinsic inverse rate constant of primary electron transfer P* (Chl *a* 802) → P^+^ (Chl *a* 802)^−^, *τ*_FL_ is the fluorescence lifetime of isolated BChl *a*, and *τ*_Q_ is the intrinsic quenching lifetime, which is assumed to be the same for both pigments BChls *a* 2 and 3 of the FMO protein.

If intradomain exciton relaxation is fast compared to interdomain energy transfer, it can be assumed that the exciton states within a domain are in quasi thermal equilibrium before interdomain transfer occurs. The interdomain energy transfer rate *k*_*a*→*b*_ can then be approximated as^41^11
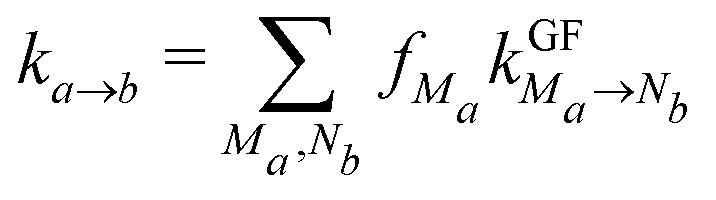
where *f*_*M*_*a*__ is the Boltzmann factor12
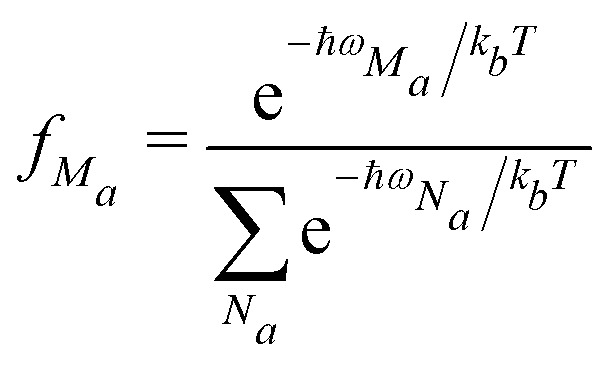


A further simplification of the interdomain rate constants^41^ can be made by combining certain domains to larger compartments and assuming fast equilibration within these compartments. The energy transfer rate constant between two such compartments *I* and *J* is given as^41^13
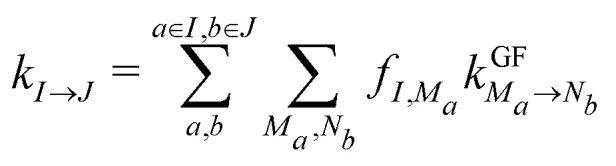
with the Boltzmann factor14
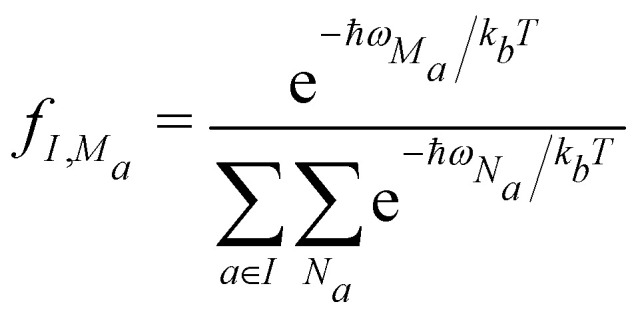
On the basis of a comparison of the different levels of coarse graining, described above, we will search for a minimal model that still captures the main characteristics of the most detailed description, in particular, the light-harvesting efficiency. In this way, it will also be possible to identify the bottlenecks of the overall light-harvesting process.

### 2.2 Parameterization of the Hamiltonian

The parameters of the Frenkel exciton Hamiltonian are obtained from structure-based calculations (excitonic couplings) and a comparison between calculated and measured optical spectra (site energies, spectral density). The excitonic couplings are calculated by employing a refined Poisson-TrEsp method^22,59,60^ that combines quantum chemical calculations of the transition densities of the isolated (fully geometry optimized) BChl *a* and Chl *a* pigments with electrostatic calculations of the screening effects caused by the electronic polarizability of the protein/solvent environment. In short, the electrostatic potential (ESP) of the transition density of the isolated pigment is fitted by the ESP of atomic transition charges (ATC). The ATCs obtained in this way are placed in molecule-shaped cavities with optical dielectric constant *ε* = 1 inside the cavities and *ε* = *n*^2^ outside, where *n* is the average refractive index of the protein/solvent environment. A Poisson equation is solved for the ESP of the transition density in the presence of a dielectric medium representing the optical polarizability of the protein/solvent environment. Details of the calculations, including the QC parts and the numeric values of the ATCs are given in the ESI,† Section S1.3.1. In order to compensate for uncertainties in the quantum chemical calculations of the overall magnitude of the transition density, the ATC are rescaled by a constant factor such that the resulting dipole moment agrees with the vacuum transition dipole moment of the pigment, inferred by extrapolation of the transition dipole moments measured in solvents with different refractive index *n*. In the present study, we use vacuum transition dipole moments of 20.2 D^2^ for Chl *a* and 43.3 D^2^ for BChl *a* as determined by Knox and Spring.^61^

In addition to the screening of the Coulomb coupling, the polarizability of the environment can lead to an enhancement of the transition density of the chromophores by reaction field effects.^62^ We have recently investigated these effects with polarizable continuum model (PCM) quantum chemical calculations^60^ on the water-soluble chlorophyll binding protein (WSCP) that contains Chl *a*. Based on these calculations, we found that the excitonic couplings are enhanced by a constant reaction field factor *f*^RF^ that has a value between 1 and 1.44. The uncertainties of the value for *f*^RF^ arises from uncertainties in the estimation of the experimental dipole strength of Chl *a* in different solvents. In addition to the reaction field effects described above, there is a polarization of the dielectric medium by the external field that causes a field enhancement in the pigment cavities. The field enhancement, also termed local field correction,^63^ still has to be determined for realistic pigment cavities. On this basis it will be possible to judge about the quality of different QC methods in the determination of the reaction field factor. So far, we have tested time-dependent density functional theory with the B3LYP exchange correlation functional and Hartree Fock configuration interaction with single excitations, where the former resulted in *f*^RF^ = 1.44 and the latter gave *f*^RF^ = 1.29.^60^ For *f*^RF^ = 1.44, the experimental dependence of the dipole strength of Chl *a* on the refractive index of the solvent can be described without taking into account local field corrections.^60^ Since the latter effects lead to an enhancement of the external field (without influencing the excitonic couplings), the value of 1.44 is an upper limit for *f*^RF^.

**Fig. 2:**
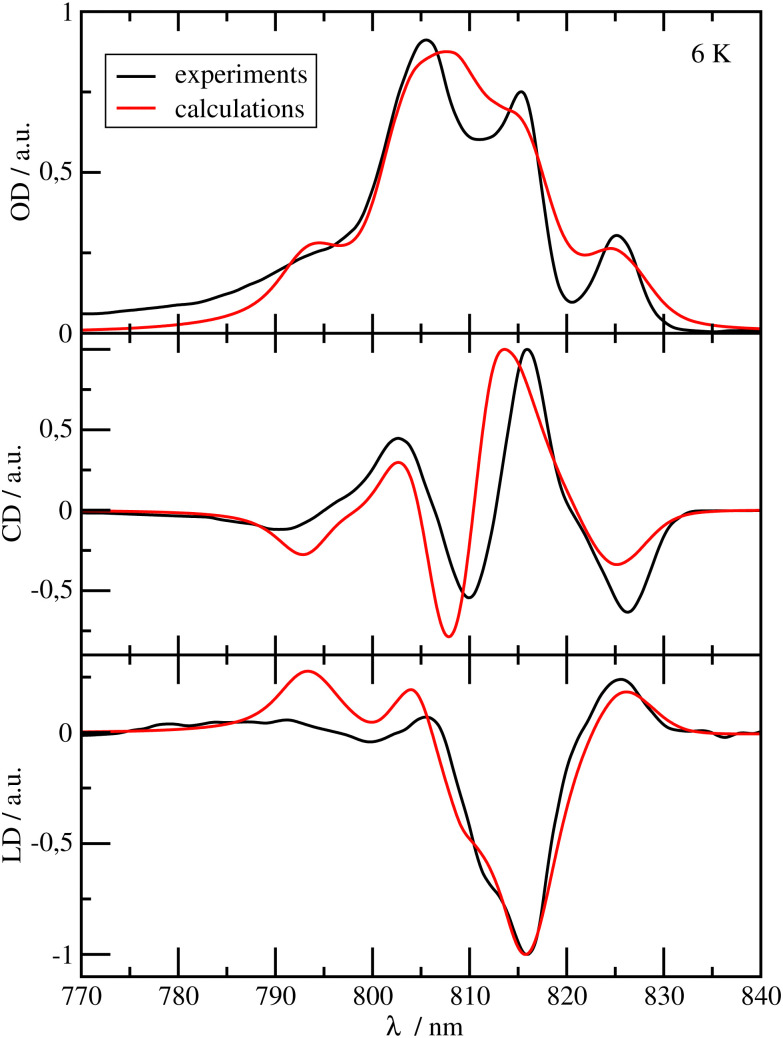
Optical spectra of the FMO protein. Comparison of experimental^24^ (black lines) linear absorption (upper panel), circular dichroism (middle panel) and linear dichroism (lower panel) spectra at *T* = 6 K with calculations (red lines), using site energies obtained from a genetic algorithm and excitonic couplings calculated with a reaction field factor *f*^RF^ = 1.15. The numerical values of the site energies and the excitonic couplings are given in the ESI,† Table S1 and Section S1.3.1, respectively.

**Fig. 3:**
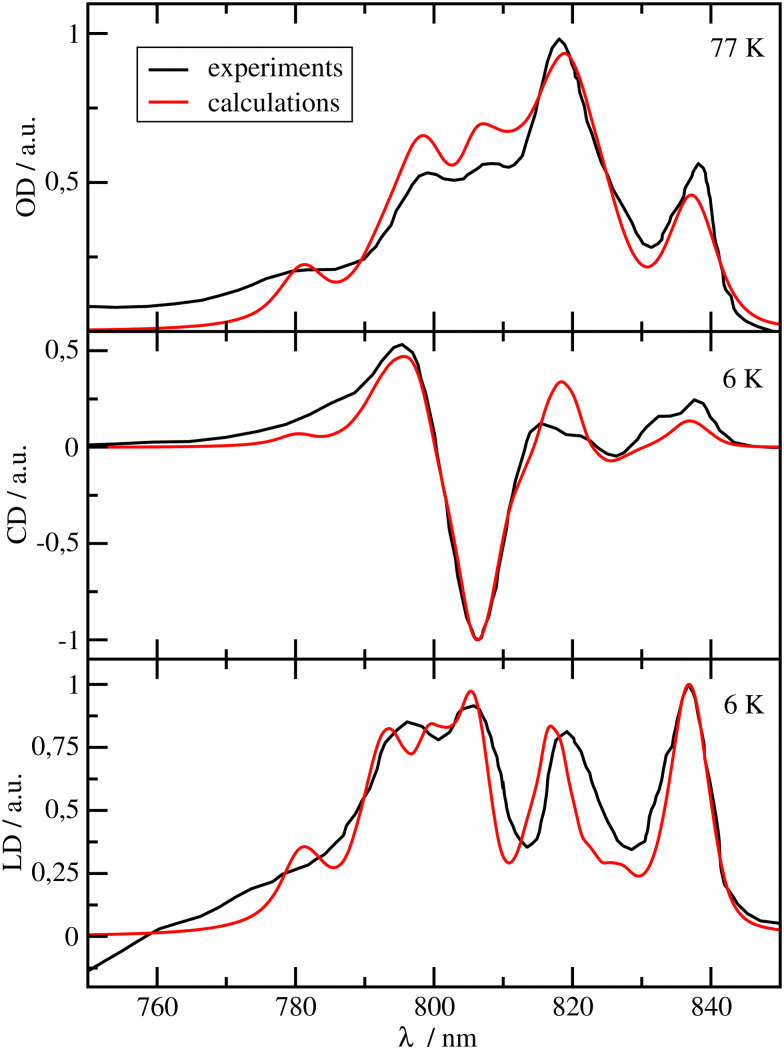
Optical spectra of the RCC. Comparison of experimental (black lines) linear absorption spectra^28^ (upper panel) at *T* = 77 K, circular dichroism (middle panel) and linear dichroism (lower panel)^51^ spectra at *T* = 6 K with calculations (red lines), using site energies obtained from a genetic algorithm and excitonic couplings calculated with a reaction field factor *f*^RF^ = 1.15. The numerical values of the site energies and the excitonic couplings are given in the ESI,† Table S2 and Section S1.3.1, respectively.

Because of the closely related structure, it is reasonable to assume the same range 1 ≤ *f*^RF^ ≤ 1.44 for BChl *a* as for Chl *a*. The uncertainty in *f*^RF^ will be taken into account in the estimate of the uncertainty of the light-harvesting efficiency. From calculations of optical spectra, described above, and comparison with experimental data, we conclude that *f*^RF^ = 1.15 is a reasonable value. The optical spectra obtained with excitonic couplings calculated using the upper limit *f*^RF^ = 1.44 fit the experimental data less (ESI,† Fig. S3), whereas the spectra calculated for the lower limit *f*^RF^ = 1.00 (ESI,† Fig. S2) give a similar agreement as those obtained for *f*^RF^ = 1.15 (Fig. 2 and 3). We took the latter value, since it is in better agreement with the QC calculations described above. Note, however, that the light-harvesting efficiencies obtained for the two *f*^RF^ values differ by only 2% (ESI,† Fig. S9).

The excitonic couplings obtained for this reaction field factor are given in the ESI,† Section S1.3.1. The largest intra-FMO monomer excitonic coupling is 85 cm^−1^ between BChls *a* 1 and 2, the largest coupling between different FMO monomers is 10 cm^−1^ (BChls *a* 2 and 5′), the largest coupling between the FMO protein and the RCC is 4 cm^−1^ (BChls *a* 3 and 807), between RCC-Ant1 and the RC it is −30 cm^−1^ (BChls *a* 801 and 811), and in the RCC it is 160 cm^−1^ between BChls *a* 801 in the special pair. Based on these couplings, it can be expected that the bottleneck for the light-harvesting is the transfer from the FMO protein to the RCC.

The mean local optical transition energies *Ē*_*m*_, termed site energies, of the pigments are obtained from a fit of the linear absorption and linear and circular dichroism spectra of the FMO protein and the RCC, using a genetic algorithm.^23^ In parallel, we have applied the quantum chemical/electrostatic charge density coupling (CDC) method^22^ to calculate the site energies directly from the structural data. Details are given in the ESI,† Sections S1.3.2 and S1.3.3.

In the calculation of optical spectra and energy transfer, we use a Gaussian distribution function to take into account static disorder in site energies, assuming the same full width at half maximum (FWHM) of 100 cm^−1^ for every site. The homogeneous spectra are averaged over 5000 realizations of static disorder in site energies. The experimental linear absorption, circular and linear dichroism spectra (OD, CD and LD, respectively) of the FMO protein, measured at 6 K, were taken from the work of Vulto *et al.*^24^ The experimental OD spectrum of the RCC at 77 K was obtained from He *et al.*^28^ and the experimental CD and LD spectra of the RCC at 6 K from Permentier *et al.*^51^

## 3 Results

### 3.1 Optical spectra

In Fig. 2, the OD, CD and LD spectra of the FMO protein calculated with the parameters discussed above, using a reaction field factor *f*^RF^ = 1.15, are compared with experimental data.^24^ The calculated spectra agree well with the experiments in particular in the low-energy (long-wavelength) region, which is most important for the energy transfer to the RCC. The relative height of the three main peaks in the OD spectrum at *λ* = 805 nm, *λ* = 815 nm and *λ* = 825 nm is reproduced very well. The OD peaks are somewhat broader in the calculations than in the experiments, whereas the widths of the calculated peaks in CD and LD are close to the experimental widths. The calculated peaks in the long wavelength half of the CD spectrum are slightly blue-shifted with respect to the experimental data. There is an excellent agreement of the calculated LD spectrum with the experiment especially in the low-energy (long-wavelength) part of the spectrum. The site energies obtained from the fit (ESI,† Table S1) are similar to those obtained by Adolphs *et al.*^23^ and Vulto *et al.*,^24^ with BChl *a* 3 being the energetically lowest pigment, followed by BChl *a* 4. The largest deviations with respect to these earlier results are obtained for the site energies of BChls *a* 5 and 6, which are interchanged as compared to the values of Adolphs *et al.*,^23^ whereas Vulto *et al.*^24^ inferred equal site energies for these pigments. In general, the site energy funnel in the FMO protein discovered earlier from fits of optical spectra for *C. tepidum*^23,24^ and *P. aestuarii*,^23,25^ from direct site energy calculations for *P. aestuarii*^21,22,26,64^ and *C. tepidum*^65^ and from site-directed mutagenesis experiments on *C. tepidum*^72^ is confirmed by the present calculations. We will investigate below, how important this funnel is for the light-harvesting efficiency of the FMO-RCC supercomplex. Please note that BChl *a* 8, which is bound at the surface of the FMO protein, was discovered later^20^ and was, therefore, not included in the earlier calculations.^21–25^ Note also, that the exact occupation of the 8th binding site is unclear and may depend on the preparation of the sample.^66^ Here, we assumed 100% occupation of this binding site. The spectra calculated with site energies from the CDC-method (using the cryo-EM structural model^7^) agree much less with experimental data (ESI,† Fig. S1) whereas those obtained with the genetic algorithm with reaction-field factor of *f*^RF^ = 1.0 and reaction-field factor of *f*^RF^ = 1.44 still provide a qualitatively correct description of the experiments (ESI,† Fig. S2 and S3). There is indeed a very weak correlation between the fitted site energies and those obtained with the structure-based CDC method (ESI,† Fig. S4).

In Fig. 3, the OD, CD and LD spectra calculated for the RCC are compared with experimental data^28,51^ revealing an overall reasonable agreement. The site energies of the RCC inferred from the fit of these spectra are given in Table S2 in the ESI.† The pigments with the lowest site energies in the RCC are BChls *a* 804 and 805 in the upper layer of the RCC-Ant subunit facing the FMO protein, the two special pair pigments BChls *a* 801 in the RC, and BChl *a* 814, which connects the lower layer of the RCC-Ant subunit to the RC (Fig. 1). The optical spectra of the RCC calculated with the CDC site energies agree much less with the experimental data (ESI,† Fig. S1) than the spectra in Fig. 3. Consequently, there is a very weak correlation between the site energies obtained from the CDC calculations and the fitted values (ESI,† Fig. S5). In the following, we will use the fitted site energies in the calculations of the light-harvesting efficiency. The CDC values will serve as a test case for the sensitivity of our results with respect to the chosen set of site energies.

The remaining deviations between the optical spectra obtained for the fitted site energies and the experimental data in Fig. 2 and 3 reflect the limitations of our theoretical description, including the following approximations: (1) assuming an equal width for the Gaussian distribution function of the static disorder in site energies for all pigments. Monte Carlo/electrostatic computations of static disorder can reveal site specific differences.^55^ (2) Assuming a homogeneous electronic polarization of the environment in the calculation of the screening of the excitonic couplings. Quantum mechanics/molecular mechanics methods have been developed to study the influence of the heterogeneous polarizability of the environment.^67^ (3) Neglecting the high-frequency intramolecular vibrations in the spectral density of the exciton–vibrational coupling. Non-perturbative line-shape theories can be used to include the respective vibronic transitions.^68,69^ Alternatively, because of their small Franck–Condon factors and, hence, excitonic couplings to other transitions, these high-frequency transitions can be included as localized transitions.^68,70^ (4) The definition of exciton domains is based on a qualitative estimate of the dynamical localization of exciton states. A non-perturbative approach could be used to explicitly describe this dynamic localization and its influence on the optical spectra and excitation energy transfer.^56^ For the present purpose, however, we consider these approximations as not critical, as will be demonstrated below by comparing light-harvesting efficiencies obtained with different sets of site energies.

### 3.2 Light-harvesting efficiency and photoprotection

The initial population of excited states was assigned by taking into account energy transfer from the baseplate to the FMO protein as described in detail previously^38^ and in the present ESI,† Section S1.1.3. From the FMO protein, the excitation energy is transferred *via* RCC-Ant1 to the RC, where it is trapped by primary electron transfer leading to the oxidized state P^+^ of the special pair. An intrinsic inverse rate constant of primary charge separation of 2 ps is assumed in the calculations. The exact value of this rate constant is not known. We assume that it is similar to values estimated for other reaction centers,^71^ which are structurally very similar. Neerken *et al.*^49^ reported a partial decay of the bleaching signal, resulting upon excitation of the primary electron acceptor Chl *a* 802 in the RC, with a time constant of 1.5 ps. Assuming ultrafast excitation energy transfer to the special pair, this time constant probably represents the most accurate estimate for the inverse rate constant of primary electron transfer in the present system. The spectral density characterizing the exciton–vibrational coupling is obtained from earlier work^23,57^ as described in the ESI,† Section S1.3.4. The intrinsic time constant *τ*_*Q*_ = 23 ps of oxidative quenching of excitation energy at BChls *a* 2 and 3 of the FMO protein has been determined such that the overall experimental fluorescence life time of 60 ps^47^ results, as described in the ESI,† Section S1.2. A fluorescence lifetime *τ*_Fl_ = 2 ns is assumed for isolated BChl *a*.

Using the parameters determined above, we calculated the exciton population dynamics at room temperature (*T* = 300 K) in models of different complexity. In the most detailed model, exciton relaxation in the exciton domains is explicitly taken into account. In the relaxed-domains model, we assume that intradomain relaxation is so fast, that interdomain transfer starts from an equilibrated excited state of the donor domain with rate constant *k*_*a→b*_ (eqn (11)). The more coarse-grained models are designed to find the bottlenecks of the light-harvesting process by integrating different exciton domains in compartments and assuming that interdomain exciton equilibration within these compartments is fast as compared to intercompartment transfer. The 5-compartment model contains the whole FMO protein, the RCC-Ant1, the RCC-RC, the RCC-Ant2 and the P^+^ compartments. In the 3-compartment model, in addition, we assume fast equilibration of excited states in the whole RCC. The 2-compartment model assumes a fast equilibration of all excited states in the FMO-RCC supercomplex.

In Fig. 1B, we compare the total population of excited states and of the state P^+^ in the different models. Normalizing the initial population of the FMO protein to unity, the equilibrium population of the charge-separated compartment of the RC (P^+^) directly gives the light-harvesting efficiency. The minimal model, that still results in the same light-harvesting efficiency and a very similar population kinetics as the complete model, is the 5-compartment model. The populations in the charge-separated compartment obtained with the complete model, the relaxed-domains model and the 5-compartment model are within 1% of each other (Fig. 1B). Assuming fast equilibration between all excited states in the RCC (3-compartment model) or between all excited states in the whole supercomplex (2-compartment model) leads to larger deviations in the population dynamics and light-harvesting efficiencies (Fig. 1B). Hence, the crucial bottlenecks of the light-harvesting process in the FMO-RCC supercomplex are the transfer from FMO to RCC-Ant1 and from the latter to the RCC-RC with average time constant of 39 ps and 23 ps, respectively (Fig. 1A). The underlying interdomain transfer time constants, which are given in ESI,† Fig. S6, show that there are two main pathways of the exciton energy transfer between the FMO protein and the RCC-Ant1 and multiple pathways between the latter and the RC. Please note that the time constants of back transfer RCC-Ant1 → FMO and RCC-RC → RC-Ant1 in the 5-compartment model are somewhat shorter with 28 ps and 11 ps, respectively, than the rate constants of forward transfer, reflecting the entropic contributions to the free energy difference from the different numbers of pigments in the compartments (Fig. 1A).

**Fig. 4:**
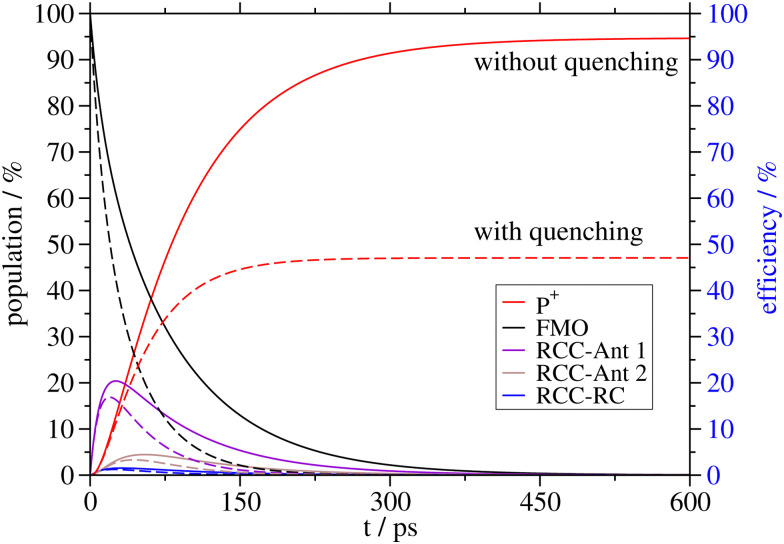
Light harvesting under anaerobic and aerobic conditions. Lightharvesting and trapping by primary charge transfer calculated in the 5-compartment model. The solid lines show the populations of the compartments under anaerobic (no quenching) conditions and the dashed lines are obtained assuming oxidative stress, where quenching of excitation energy occurs in the FMO protein, as described in the text.

The populations of compartments obtained in the 5-compartment model under aerobic (oxidative quenching in the FMO protein) and anaerobic (no quenching) conditions are compared in Fig. 4. The light-harvesting efficiency of 95% for anaerobic conditions drops to 47% in the presence of oxidative quenching in the FMO protein. Because the transfer between RCC-Ant1 and RCC-RC is somewhat faster than the transfer from the FMO protein to RCC-Ant1, the population of the excited states in the latter does not exceed 21% of the overall excitation in the system. The population of RCC-Ant2 is much smaller because of the fast electron transfer in the RC that traps the excitation energy arriving from RCC-Ant1 before it can be transferred to RCC-Ant2. We will concentrate in the following on anaerobic conditions (no quenching) and investigate first different factors that influence the light harvesting efficiency. Afterwards we study the sensitivity of our results with respect to the uncertainties of the parameters in our model.

**Fig. 5:**
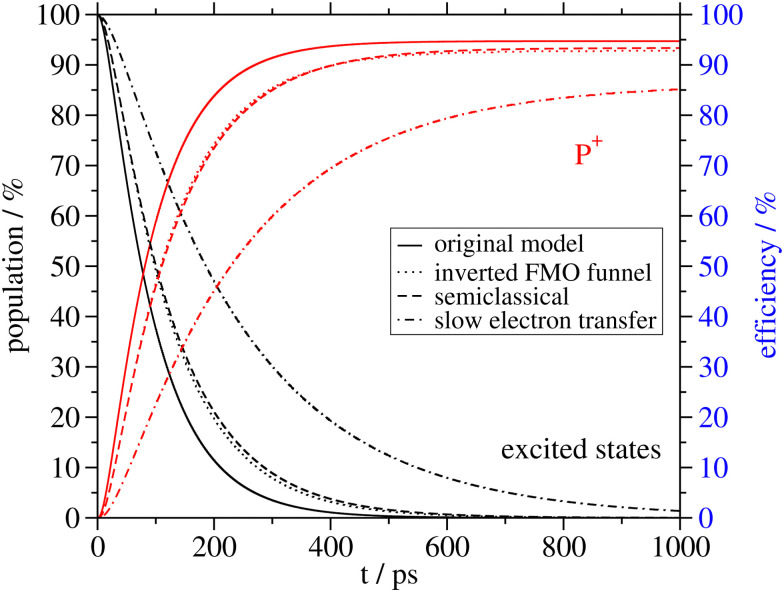
Different factors affecting the light-harvesting efficiency. Population of excited states (black lines) and charge separated state P^+^ obtained in the 5-compartment model with the original parameters are compared to those obtained by inverting the site energy funnel in the FMO protein, a classical description of nuclear motion, and assuming a slow electron transfer as explained in the text.

The present calculations allow to estimate the significance of the site energy funnel in the FMO protein^21–26,72^ for the light-harvesting efficiency of the supercomplex. For this purpose, we have inverted this funnel by calculating a mean site energy *Ē* of all pigments in the FMO protein and inverting the sign of all site energy shifts Δ*E*_*m*_ = *Ē*_*m*_ − *Ē*, such that the new site energy 
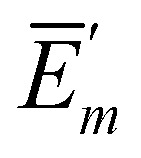
 is given as 
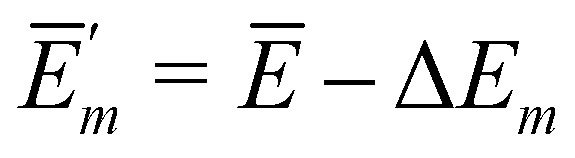
. This inversion is found to increase the time constant of FMO → RCC energy transfer about two-fold from 39 ps to 73 ps. Nevertheless, the light-harvesting efficiency decreases by only 2% from 95% with the site energy funnel to 93% with the inverted funnel (Fig. 5). This small change reflects the fact that the new transfer time constant between FMO and RCC is still small compared to the fluorescence lifetime of 2 ns. Slowing down primary electron transfer by assuming an inverse intrinsic rate constant of 25 ps instead of 2 ps, used so far, reduces the light-harvesting efficiency by about 10% (Fig. 5). In this case, part of the excitation energy can escape from the RC, which is seen in the much larger population of the RCC-Ant2 subunit (ESI,† Fig. S7), as compared to the case of fast electron transfer (Fig. 4).

Using a classical description of nuclear motion, that is, applying the semiclassical expression for the generalized Förster rate constants and the Redfield rate constants, results in an efficiency loss of about 1.5% (Fig. 5). This loss is due to the semiclassical Redfield theory rate constants, leading to equal equilibrium populations of all exciton state in a given domain.^38^ The semiclassical generalized Förster theory rate constants are even slightly larger than their quantum equivalents (Fig. S8, ESI†), and obey the principle of detailed balance.^40,45^ The efficiencies of excitation energy trapping obtained for the different cases in the 5 compartment model in Fig. 5 demonstrate that a slow electron transfer affects the light-harvesting efficiency much stronger than an inverted site energy funnel in the FMO or a classical description of nuclear motion.

In the following, we study the sensitivity of the calculated efficiencies on the parameters used. For the two limits of the reaction field factor, *f*^RF^ = 1 and *f*^RF^ = 1.44, the efficiencies vary between 92% and 97%, respectively under anaerobic (no quenching) conditions and between 35% and 65% under oxidative stress (ESI,† Fig. S9). Note that for every set of excitonic couplings, we have re-optimized the site energies from a fit of optical spectra (ESI,† Fig. S2 and S3). In addition, we have performed calculations for different sets of site energies using excitonic couplings obtained for our optimal reaction field factor *f*^RF^ = 1.15 and assuming anaerobic conditions (no oxidative quenching). Despite the very weak correlations between the structure-based site energies, obtained with the CDC method, and the fitted site energies (ESI,† Fig. S4 and S5), the light-harvesting efficiencies obtained with these two sets of site energies are practically identical, close to 95% (ESI,† Fig. S10). If in the set of fitted site energies, those of the pigments in RCC-Ant1 and RCC-Ant2 are chosen to be equal (12 500 cm^−1^) the light-harvesting efficiency drops by 2% to 92% (ESI,† Fig. S10). Including a second FMO trimer bound to the homodimeric RCC at a symmetric position to the first FMO trimer has practically no influence on the light-harvesting efficiency (ESI,† Fig. S11). From the above calculations, we estimate that the light-harvesting efficiency of the FMO-RCC supercomplex is (95 ± 3)% under anaerobic conditions.

### 3.3 Robustness

**Fig. 6:**
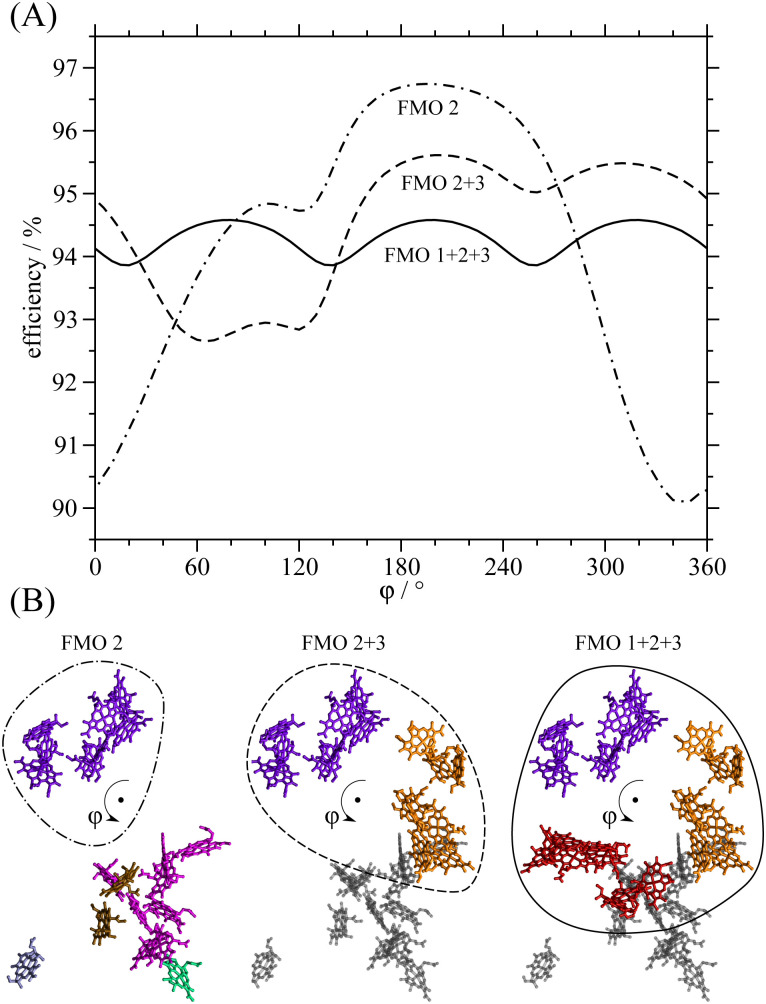
Robustness of light harvesting. (A) Energy transfer efficiency calculated for different fictitious situations, in which the FMO protein is monomeric (FMO 2), dimeric (FMO 2 + 3) or trimeric (FMO 1 + 2 + 3) and it is rotated around the symmetry axis of the trimer by an angle *φ* relative to the RCC. (B) Illustration of the configurations considered in (A). Only the RCC-Ant1 subunit of the RCC is shown and for FMO 2 + 3 and FMO 1 + 2 + 3 the RCC-Ant1 is shown in grey for better visibility.

Next, we study how robust the excitation energy transfer is with respect to changes in the docking interface between the FMO protein and the RCC. For this purpose, we consider a rotation of the FMO trimer along its symmetry axis. In order to investigate the role of the *C*_3_ symmetry of the FMO protein, we also study how the light-harvesting efficiency changes if one or two FMO monomers are removed. The bottom of Fig. 6 shows the three different cases considered. In the “FMO 1 + 2 + 3” model the orientation of the trimer with respect to the RCC is varied, in “FMO 2 + 3” and “FMO 2” we consider hypothetical dimeric and monomeric FMO proteins, respectively, that are also rotated around the *C*_3_-symmetry axis of the trimer. The top part of Fig. 6 shows the dependence of the light-harvesting efficiency on the rotation angle *φ*, calculated with the three models. For the FMO trimer (FMO 1 + 2 + 3), the efficiency varies by less than 1% around 94.3%, with a periodicity of 120° reflecting the *C*_3_ symmetry of the FMO protein. Removing monomer 1 (FMO 2 + 3), the efficiency at the native orientation (*φ* = 0) is slightly higher than for the trimer. This result is due to the fact that the total excitation energy is now only distributed between 2 monomers. A minimum efficiency of 92.5% is reached at *φ* = 60° and a maximum of 95.5% occurs at *φ* = 200°. Monomer 2 is most distant from RCC at the native orientation *φ* = 0. Thus removing monomers 1 and 3 (FMO 2), the efficiency at *φ* = 0 is almost at its minimum at 90%. However, rotating by 180° brings monomer 2 closest to the RCC increasing the efficiency to 96.5%. This is the highest value obtained, since the excitation energy is now concentrated at only one FMO monomer. In any of these models, the efficiency does not drop below 90% for any given rotation.

## 4 Discussion

Taking into account that a typical chlorosome of *C. tepidum* contains 200 000 BChl *c* molecules^17^ and that 3% of all BChl pigments in *C. tepidum* cells are BChl *a*^73^ and 1% of all BChls in the chlorosomes are BChl *a* in the baseplate,^74^ we have about 4000 BChl *a* pigments bound in the RCC complexes connected to one chlorosome. Assuming two FMO trimers bound to every RCC, there are 74 BChl *a* per RC and hence 4000/74 ≈ 50 RC connected to one chlorosome. Thus, every RC receives excitation energy from roughly 200 000/50 = 4000 BChl pigments. This number is larger by one order of magnitude than in any other photosynthetic light-harvesting apparatus on earth. It seems obvious that GSB cannot afford to lose efficiency in the final step of excitation energy transfer. The present calculations, indeed, provide compelling evidence that light harvesting in the FMO-RCC supercomplex under anaerobic (non-quenching) conditions occurs with an efficiency of about (95 ± 3)%. Uncertainties in our efficiency estimate concern the exact values of the excitonic couplings and site energies. The first high-resolution cryo-EM structure^7^ of the FMO-RCC supercomplex revealed only one FMO protein bound to the RCC, whereas the second study^33^ found the second FMO trimer. Obviously, the binding of the FMO protein to the RCC is rather weak and, therefore, a preparation of FMO-RCC supercomplexes is not an easy task. In the light of the weak binding, it might also happen that the FMO protein attaches in a different orientation to the membrane containing the RCC. The present calculations demonstrate that the *C*_3_ symmetry of the FMO protein makes the energy transfer very robust against such variations. For the second FMO trimer,^33^ the closest pigment–pigment distance to the RCC is comparable to the one found in the other FMO trimer, although the latter has a pigment in two FMO monomers in close proximity to the RCC complex, whereas the former has only one such monomer. Noting that energy transfer between different FMO monomers, occurring with a time constant of about 10 ps (ref. 45 and ESI,† Fig. S6) is four times faster than the transfer between the FMO protein and the RCC (Fig. 1A), we expect a similar light-harvesting efficiency of both FMO trimers.

The present system offers the chance to evaluate different factors contributing to the high light-harvesting efficiency. The site energies obtained from a fit of optical spectra of the FMO and RCC subunits reveal low-energy sites at the interfaces between these complexes and of the primary electron donor, the special pair in the RCC. The location of low-energy sites in the RCC-Ant complex increases the efficiency of light harvesting by about 1% as compared to the case of equal site energies in these subcomplexes. Using a classical description of nuclear motion in the whole FMO-RCC supercomplex reduces the light-harvesting efficiency by 1.5%. This effect is caused by the equal equilibrium populations of the exciton states in the different domains that effectively decrease the population of exciton states that are localized at the interface of the different subunits. Inverting the site energy funnel in the FMO protein, within a quantum description of nuclear motion, moves exciton state populations from the interface to the RCC towards the baseplate. Nevertheless, the light-harvesting efficiency just decreases by 2%. Such small changes at the first glance do not look critical. However, under extreme light conditions as experienced by GSB, small changes could still provide important evolutionary advantages.

In the photosystems of higher plants, no such site energy funnels are known. Instead, one finds low-energy sites far away from the reaction centers, as, *e.g.*, in the case of the core complex of photosystem II^41^ and the peripheral LHCI light-harvesting complex of photosystem I.^2^ Obviously, these systems can afford to lose a few percent of light-harvesting efficiency, since under normal light conditions, the aspect of photoprotection becomes more important. However, one common principle for efficient light-harvesting of GSB and other photosynthetic organisms seems to be a fast primary charge transfer, needed to beat the entropic penalty of decreasing the free energy of the antennae relative to the RC because of the larger number of antennae states. Slowing down primary electron transfer by a factor of 10 results in an efficiency loss of roughly 10% in the present system. The present time constant of 23 ps obtained for the transfer between the antenna compartment RCC-Ant1 and the RC fits nicely the 25 ps time constant reported for the decay of excited states in the RCCs by time-resolved spectroscopy.^49^ Because of the lack of structural information, this decay has been interpreted in terms of a trapping limited^12,49^ as well as a transfer-to-the-trap limited model.^12,49,50^ The present calculations strongly suggest that the latter model is correct.

Despite the small light intensities experienced by GSB, there is still a need to protect the RC of GSB from excitation energy under certain conditions. If molecular oxygen is present, the latter is known to react with reduced Fe–S electron acceptors damaging the RCC.^47^ The present calculations show that the protective quenching mechanism of excited BChls *a* 2 and 3 states of the FMO protein, inferred from site directed mutagenesis experiments,^47^ reduces the light-harvesting efficiency by about (50 ± 15)%, where the uncertainty arises from the uncertainty of the reaction field factor entering the calculation of excitonic couplings. This result suggests that the small light-harvesting efficiency of FMO-RCC supercomplexes reported in the literature could also be caused by oxidative quenching in the FMO protein. It is also worth noting that the quenching is much more efficient at BChl 3 than at BChl 2, because of the higher equilibrium population of the former (ESI,† Fig. S12). This finding is in agreement with the fact that in the experiment, a much stronger oxidative quenching of the fluorescence is observed for the C49A mutant that lacks Cys 49 at BChl 2 than for the C353A mutant lacking Cys 353 at BChl 3.^47^ In our calculations, the light-harvesting efficiency is reduced from 94% under anaerobic conditions to 80% and 48% if the quenching occurs only at BChl 2 or BChl 3, respectively (ESI,† Fig. S13). In the presence of both quenchers, the efficiency further decreases by only 1% to 47% as compared to the latter case. Obviously, Cys 353 in the neighborhood of BChl 3 is much more important for photoprotection than Cys 49 at BChl 2. Indeed, the former is conserved across all green sulfur bacteria, whereas the latter only occurs in some members.^47^

In summary, we have shown that the light-harvesting efficiency of the FMO-RCC supercomplex is about (95 ± 3)%, a value that is much higher than reported experimentally. Most likely, in the experimental preparation, a certain fraction of FMO proteins got disconnected from the RCC or was in a quenched state. Our calculations show that the exact orientation between FMO protein and RCC is not critical for the excitation energy transfer, as long as both complexes stay connected. Inverting the site energy funnel in the FMO protein decreases the efficiency by roughly 2%. Quantum effects of nuclear motion are found to be responsible for about 1% of the light-harvesting efficiency. Primary electron transfer in the RC is fast compared to the transfer between core antenna and the RC in the RCC, that is, light-harvesting in the RCC is transfer-to-the trap limited. The latter aspect has the most critical influence on the light-harvesting efficiency under anaerobic (non-quenching) conditions.

## Author contributions

A. K. performed the calculations of optical spectra and energy transfer, D. L. and F. M. performed the quantum chemical and electrostatic calculations, A. K. and T. R. wrote the manuscript with input from all coauthors. T. R. supervised the project. All authors read and approved the final version of the manuscript.

## Conflicts of interest

There are no conflicts to declare.

## Supplementary Material

CP-025-D3CP01321A-s001
