# Supplementary material for: Living on the edge: light-harvesting efficiency and photoprotection in the core of green sulfur bacteria
Source: Phys Chem Chem Phys. 2023 Jun 26;25(28):18698–710. doi: 10.1039/d3cp01321a (PMC10355171; doi:10.1039/d3cp01321a)
Supplement: CP-025-D3CP01321A-s001 [file CP-025-D3CP01321A-s001.pdf]

Electronic Supplementary Material (ESI) for Physical Chemistry Chemical Physics.
This journal is © the Owner Societies 2023

Electronic Supplementary Information

Living on the edge: Light-harvesting efficiency and photoprotection in the core of green sulfur bacteria

Alexander Klinger, Dominik Lindorfer, Frank Müh, and Thomas Renger

Institut für Theoretische Physik, Johannes Kepler Universität Linz, Altenberger Str. 69, 4040 Linz, Austria.
E-mail: thomas.renger@jku.at

S1 Theory

S1.1 Rate constants of energy transfer

S1.1.1 Intradomain excitation energy transfer: Redfield theory

For the relaxation of excitons within a domain of strongly coupled pigments, we employ Redfield theory. The relaxation rate constant between the exciton states $|M_a\rangle$ and $|N_a\rangle$ in domain a is given as[1]

$$\begin{aligned} k^{\mathrm{Redf}}_{M_a \to N_a} &= 2\pi\omega^2_{M_a N_a} \sum_{m_a} \left| c^{(M_a)}_{m_a} \right|^2 \left| c^{(N_a)}_{m_a} \right|^2 \\ &\times \quad \{ J(\omega_{M_a N_a})(1 + n(\omega_{M_a N_a})) + J(\omega_{N_a M_a}) n(\omega_{N_a M_a}) \} \end{aligned} \tag{S1}$$

with the Bose-Einstein distribution function

$$n(\omega) = \frac{1}{e^{\hbar\omega/k_{\mathrm{B}}T} - 1} \tag{S2}$$

the transition frequency $\omega_{K_a L_a} = \omega_{K_a} - \omega_{L_a}$ between exciton states $|K_a\rangle$ and $|L_a\rangle$, and the spectral density $J(\omega) = \sum_\xi g^2_\xi \delta(\omega - \omega_\xi)$ that contains the local coupling constants (eq 1 of the main text), which are assumed to be independent of the site index m_a. Note that we have investigated this approximation in a recent normal mode analysis of the spectral density of FMO trimers[2] and found that although the local coupling constants can vary up to a factor of two, these variations have practically no influence on energy transfer.

S1.1.2 Interdomain excitation energy transfer: Generalized Förster theory

The excitation energy transfer between different domains is described by generalized Förster theory.[3–7] Assuming uncorrelated fluctuations of site energies in different domains, as investigated by a recent normal mode analysis of the spectral density,[2] the rate constant in generalized Förster theory $k^{\mathrm{GF}}_{M_a \to N_b}$ between states $|M_a\rangle$ and $|N_b\rangle$ of domain a and b, respectively, is given in eq 6 of the main text. It contains the overlap integral between the normalized absorption lineshape function $D_{N_b}(\omega)$ of the acceptor exciton state and the normalized fluorescence lineshape function $D'_{M_a}(\omega)$ of the donor exciton state, which are given as[8]

$$D_{N_b}(\omega) = \frac{1}{2\pi} \int_{-\infty}^{\infty} dt e^{-i(\omega - \tilde{\omega}_{N_b})t} e^{G_{N_b}(t) - G_{N_b}(0)} e^{-|t|/\tau_{N_b}} \tag{S3}$$

and

$$D'_{M_a}(\omega) = \frac{1}{2\pi} \int_{-\infty}^{\infty} dt e^{i(\omega - \tilde{\omega}_{M_a})t} e^{G_{M_a}(t) - G_{M_a}(0)} e^{-|t|/\tau_{M_a}} \tag{S4}$$

respectively, where $V_{M_a N_b}$ is the inter-domain excitonic coupling given in eq 5 of the main text. The function $G_{M_a}(t)$ reads

$$G_{M_a}(t) = L^{-1}_{M_a} \int_{-\infty}^{\infty} d\omega \left\{ (1 + n(\omega)) e^{-i\omega t} + n(\omega) e^{i\omega t} \right\} J(\omega) \tag{S5}$$

with the inverse participation ratio

$$L^{-1}_{M_a} = \sum_{m_a} \left| c^{(M_a)}_{m_a} \right|^4 \tag{S6}$$

of exciton state $|M_a\rangle$, the $n(\omega)$ from eq S2, and the spectral density $J(\omega)$ of the site energy fluctuations. The exciton relaxation-induced inverse dephasing time $\tau^{-1}_{M_a}$ is given as

$$\tau_{M_a}^{-1} = \frac{1}{2} \sum_{N_a}^{N_a \neq M_a} k_{M_a \to N_a}^{\mathrm{Redf}} \tag{S7}$$

and the shifted transition frequencies are defined as

$$\tilde{\omega}_{M_a N_b} = \tilde{\omega}_{M_a} - \tilde{\omega}_{N_b} \tag{S8}$$

with

$$\tilde{\omega}_{K_c} = \omega_{K_c} - E_\lambda^{(K_c)}/\hbar + \Delta\omega_c \tag{S9}$$

Here, ω_{K_c} is the vertical excitation frequency of exciton state $|K_c\rangle$ in domain c as given in eq 3 of the main text and $E_\lambda^{(K_c)}$ is the reorganization energy due to the diagonal elements of the exciton-vibrational coupling of this exciton state, reading

$$E_\lambda^{(K_c)} = L_{K_c}^{-1} E_\lambda^{(\mathrm{loc})} \tag{S10}$$

with the inverse participation ratio of exciton state $|K_c\rangle$ (eq S6) and the reorganization energy $E_\lambda^{(\mathrm{loc})}$ of the local pigment excitation, given in eq 2 of the main text. The $\Delta\omega_c$ in eq S9 is a small additional transition energy shift caused by the off-diagonal elements of the exciton-vibrational coupling

$$\Delta\omega_c = \sum_{M_c}^{M_c \neq K_c} \sum_{m_c} \left| c_{m_c}^{(K_c)} \right|^2 \left| c_{m_c}^{(M_c)} \right|^2 \wp \int_{-\infty}^{\infty} d\omega \frac{\omega^2 \left\{ (1 + n(\omega)) J_{m_c}(\omega) + n(-\omega) J_{m_c}(-\omega) \right\}}{\omega_{K_c M_c} - \omega} \tag{S11}$$

where $\wp$ denotes the principal part of the integral.

Treating the motion of the nuclei classically, the semi-classical rate constant $k_{M_a \to N_b}^{\mathrm{scGF}}$ is obtained as[2]

$$k_{M_a \to N_b}^{\mathrm{scGF}} = \frac{|V_{M_a N_b}|^2}{\hbar} \sqrt{\frac{\pi}{k_{\mathrm{B}} T E_\lambda^{(M_a,N_b)}}} \exp\left\{ -\frac{(\hbar\tilde{\omega}_{M_a N_b} - E_\lambda^{(M_a,N_b)})^2}{4 k_{\mathrm{B}} T E_\lambda^{(M_a,N_b)}} \right\} \tag{S12}$$

where T is the temperature, $\tilde{\omega}_{M_a,N_b}$ is given in eq S8, $V_{M_a N_b}$ is the interdomain exciton coupling (eq 5), and it holds that $E_\lambda^{(M_a,N_b)} = E_\lambda^{(M_a)} + E_\lambda^{(N_b)}$ in the present limit of uncorrelated site energy fluctuations with the $E_\lambda^{K_c}$ in eq S10.

S1.1.3 Exciton population dynamics and initial conditions

The master equations eq 7 of the main text can be written in matrix form as $\frac{\mathrm{d}}{\mathrm{d}t}\boldsymbol{P}(t) = -\hat{A}\boldsymbol{P}(t)$, with the kinetic matrix $\hat{A}$. This system of ordinary differential equations has the solution

$$\boldsymbol{P}(t) = \sum_i d_i \boldsymbol{c}_i \mathrm{e}^{-\lambda_i t} \tag{S13}$$

where $\boldsymbol{c}_i$ and λ_i are the eigenvectors and eigenvalues of the matrix $\hat{A}$, respectively. The constants d_i are obtained from the initial conditions $\boldsymbol{P}(0) = \sum_i d_i \boldsymbol{c}_i$ and $\boldsymbol{P}(0)$ denotes the initial population of the FMO-RCC. For the initial population of the system, we assume incoherent excitation energy transfer from the baseplate to the FMO protein and no direct energy transfers from the baseplate to the RCC. The initial population of an exciton state in the FMO protein is assumed to be proportional to the rate constant for the transfer from the baseplate[9]

$$P_{M_a}^{(a)}(0) \propto |V_{bM_a}|^2 \int \mathrm{d}\omega D_b'(\omega) D_{M_a}(\omega) \tag{S14}$$

where V_{bM_a} is the electronic coupling between the lowest exciton state in the baseplate and the Mth exciton state in domain a in the FMO protein. Taking into account that the transition dipole moment of the lowest energy exciton state of the baseplate is oriented in the plane of the baseplate[10] and, therefore, normal to the symmetry axis $\boldsymbol{e}_{\mathrm{FMO}}$ of the FMO protein, the excitonic coupling V_{bM_a} is estimated to be

$$V_{bM_a} \propto \frac{\sin\theta_{M_a}}{\left(R_{0a} + \Delta R_{M_a}\right)^3} \,. \tag{S15}$$

Here, θ_{M_a} is the angle between $\boldsymbol{e}_{\mathrm{FMO}}$ and the transition dipole moment $\boldsymbol{\mu}_{M_a}$, R_{0a} is the vertical distance between the center of pigment 8 in domain a and the baseplate. ΔR_{M_a} takes into account the delocalization of the exciton state $|M_a\rangle$, it reads

$$\Delta R_{M_a} = \frac{1}{N}\left(\sum_{m_a}^{N,|c_{m_a}^{M_a}|^2>0.3} \boldsymbol{R}_{m_a} - \boldsymbol{R}_{8a}\right)\cdot \boldsymbol{e}_{\mathrm{FMO}} \tag{S16}$$

where $\boldsymbol{R}_{m_a}$ is the center of pigment m in domain a. The sum in eq S16 takes into account only those pigments m_a with an exciton coefficient that fulfills the condition $\left|c_{m_a}^{(M_a)}\right| > 0.3$. We normalize the initial population, that is, $|\boldsymbol{P}(0)| = 1$.

S1.2 Estimation of the intrinsic quenching time constant

If the FMO protein is under oxidative stress, the measured fluorescence rate constant is $\langle k_{fl}\rangle = 60\,\mathrm{ps}^{-1}$.[11] The inverse rate constant is calculated as

$$\langle k_{\mathrm{fl}}\rangle = \left\langle \sum_M f_M \left(k_{M\to g} + k_{M\to Q}\right)\right\rangle \tag{S17}$$

where f_M is the Boltzmann factor, $k_{M\to g}$ is the transition rate from the Mth excited state into the ground state, $k_{M\to Q}$ is the quenching rate constant, and $\langle\dots\rangle$ denotes an average over static disorder in site energies. Because $k_{M\to g}$ is in the order of ns, it can safely be neglected. Therefore, we can write

$$\langle k_{\mathrm{fl}}\rangle \approx \left\langle \sum_M f_M k_{M\to Q}\right\rangle = \left\langle \sum_M f_M \left(\left|c^{(M)}_{\mathrm{BChl\,a\,2}}\right|^2 + \left|c^{(M)}_{\mathrm{BChl\,a\,3}}\right|^2\right)\right\rangle k_{\mathrm{intr}} \tag{S18}$$

where k_{intr} is the intrinsic quenching rate constant, $c^{(M)}_{\mathrm{BChl\,a\,2}}$ and $c^{(M)}_{\mathrm{BChl\,a\,3}}$ are the exciton coefficients of the BChls a 2 and 3, respectively, in the Mth exciton state in an FMO monomer. From our calculations, the intrinsic rate constant is estimated to be $k_{\mathrm{intr}} = (22.55\,\mathrm{ps})^{-1}$.

S1.3 Parameters

S1.3.1 Excitonic couplings: The refined Poisson Transition Charges from Electrostatic Potential (TrEsp) method

Every pigment is described by a molecule-shaped cavity containing the atomic partial charges determined from a fit of the ab-initio electrostatic potential (ESP) of the transition density in vacuum. Details of the quantum chemical calculations are given below (section S1.3.3).

For each pigment m_a, the Poisson equation

$$\nabla\cdot\left(\varepsilon(\boldsymbol{r})\nabla\phi_{m_a}(\boldsymbol{r})\right) = -4\pi\sum_I q_I^{(m_a)}\delta\left(\boldsymbol{r}-\boldsymbol{R}_I\right) \tag{S19}$$

is solved numerically using MEAD,[12] revealing the ESP of the transition density of pigment m_a in a heterogeneous dielectric environment. The optical dielectric constant $\varepsilon(\boldsymbol{r}) = 1$ in the cavity and $\varepsilon(\boldsymbol{r}) = 2$ in the protein/solvent

environment. Using the electrostatic potential $\phi_m(\boldsymbol{r})$, the excitonic coupling V_{m_a,n_b} between pigment m in domain a and pigment n in domain b is given as

$$V_{m_a,n_b} = f^{\mathrm{RF}} \sum_I q_I^{(n_b)} \phi_{m_a} \left(R_I^{(n_b)} \right) \tag{S20}$$

where $q_I^{(n_b)}$ is the transition charge of atom I in pigment n_b, $\phi_{m_a}\left(R_I^{(n_b)}\right)$ is the electrostatic potential of the transition charges of pigment m_a at the position of the Ith atom in pigment n_b, and f^{RF} is the reaction field factor that takes into account an enhancement of the transition density of the chromophores by the polarization of the environment.[13] The excitonic couplings obtained for $f^{\mathrm{RF}} = 1.15$ can be found in the accompanying file "exc_coupling.dat".

S1.3.2 Site energies: The Charge Density Coupling (CDC) method

In the CDC-method[14], the charge density of the ground and excited state of the pigments is approximated by atomic partial charges. These atomic partial charges are obtained from a fit of the ab-initio ESP, as described below. The site energy E_{m_a} of pigment m in domain a is obtained as $E_{m_a} = E_0 + \Delta E_{m_a}$, where E_0 is a reference energy and ΔE_{m_a} is the site energy shift. The site energy shift in the CDC method is then given as[14]

$$\Delta E_{m_a} = \frac{1}{\varepsilon_{\mathrm{eff}}} \sum_{I,J} \frac{\left(q_I^{(m_a)}(1,1) - q_I^{(m_a)}(0,0) \right) q_J(0,0)}{\left| \boldsymbol{R}_I^{(m_a)} - \boldsymbol{R}_J \right|} \tag{S21}$$

where the sum over I runs over all partial charges of pigment m_a, the sum over J runs over all ground state background charges $q_J(0,0)$, and $q_I^{(m_a)}(0,0)$ and $q_I^{(m_a)}(1,1)$ are the ground and excited state partial charges of pigment m_a, respectively. $\boldsymbol{R}_I^{(m_a)}$ and $\boldsymbol{R}_J$ are the positions of the Ith atomic partial charge of pigment m_a and the Jth atomic partial charge of the background, respectively, and $\varepsilon_{\mathrm{eff}}$ is an effective dielectric constant, which takes into account screening and polarization effects.

Table S1: Site energies of the FMO protein in units of cm^{-1} obtained from fits of optical spectra of the FMO protein (Figs. 2, S2 and S3). Columns 2 - 4 contain the values obtained by using three different reaction field factors f^{RF} in the calculations of the excitonic couplings. Columns 5 and 6 contain earlier estimates from the literature. Please note that, pigment 8 was discovered later[15,16] and, therefore, no site energy value for this pigment was obtained in Refs.[17] and[18].

	f^{RF}			literature	
pigment	1.0	1.15	1.44	Adolphs[17]	Vulto[18]
BChl *a* 1	12360	12430	12440	12410	12400
BChl *a* 2	12410	12550	12530	12530	12600
BChl *a* 3	12160	12176	12186	12210	12140
BChl *a* 4	12270	12280	12294	12320	12280
BChl *a* 5	12580	12550	12570	12480	12500
BChl *a* 6	12500	12490	12507	12630	12500
BChl *a* 7	12420	12455	12452	12440	12430
BChl *a* 8	12410	12355	12300	-	-

S1.3.3 Quantum chemical calculations

The ab-initio charge density of the ground (S_0) and the first excited (S_1) states of BChl *a* and Chl *a* were obtained with (time-dependent) density functional theory ((TD)DFT) using Q-Chem[20]. The geometries of the isolated BChl *a* and Chl *a* pigments were optimized with DFT using the B3LYP exchange-correlation (XC) functional and a 6-31G(d,p) basis set. Based on this geometry, the ground and excited state charge densities and the transition

Table S2: Site energies of the RCC in units of cm^{-1} obtained from fits of optical spectra of RCC (Figs. 3, S2 and S3). Columns 2 - 4 contain the values obtained by using three different reaction field factors f^{RF} in the calculations of the excitonic couplings. The numbering of the pigments is according to Chen *et al.*[19]. The site energies of the pigments in the second monomer of RCC are assumed to have the same values as their symmetry equivalents in the first monomer.

	f^{RF}		
pigment	1.0	1.15	1.44
BChl *a* 801	12099	12137	12196
Chl *a* 802	15008	15004	15008
Chl *a* 803	15096	15093	15096
BChl *a* 804	12024	12020	12001
BChl *a* 805	12256	12195	12235
BChl *a* 806	12626	12616	12610
BChl *a* 807	12338	12429	12269
BChl *a* 808	12278	12455	12507
BChl *a* 809	12288	12270	12295
BChl *a* 810	12554	12526	12518
BChl *a* 811	12749	12747	12625
BChl *a* 812	12462	12465	12529
BChl *a* 813	12579	12522	12578
BChl *a* 814	12280	12235	12234
BChl *a* 815	12220	12286	12176

densities between those two states were calculated with (TD)DFT using the CAM-B3LYP XC-functional and a 6-31G(d,p) basis set. The fit of the ESP of the ground and excited state charge densities and the transition densities was performed with CHELP-BOW[21]. The resulting atomic partial charges $q_k(0)$ and $q_k(1)$ of the ground and excited state, respectively, are given in Table S3 (Chl *a*) and in the SI of Ref.[2] (BChl *a*). The atomic transition charges $q_k(0,1)$ can be found in Tabs. S4 (Chl *a*) and S5 (BChl *a*).

S1.3.4 Spectral Density

In our earlier work[2,22], we developed a microscopic model to calculate the intermolecular part of the spectral density of pigment-protein complexes (PPCs) using a combination of TrEsp/CDC with a normal mode analysis of the whole PPC. Our applications to FMO monomers[22] and trimers[2] revealed correlations in the site energy fluctuations, differences in Huang-Rhys factors between sites, the relative strength of fluctuations in site energies and excitonic couplings, and the importance of intermolecular and intramolecular (taken from experimental data) parts of the spectral density. In summary, the excitonic couplings were found to fluctuate much less than the site energies, and the correlations in site energy fluctuations have practically no influence on intradomain exciton relaxation or interdomain excitation energy transfer. The difference in local Huang-Rhys factors of the pigments is also not critical for energy transfer and both the inter- as well as intramolecular parts of the spectral density are important. Based on these results we use the following approximations: (i) neglect of fluctuations in excitonic couplings, (ii) neglect of correlations in site energy fluctuations, (iii) assuming a site-independent spectral density. The latter has been obtained from fluorescence line narrowing spectra of the B777 complex[8] and later applied to many different PPCs[7,17,23–25], including the FMO protein[17]. This spectral density reads

$$J(\omega) = S J_0(\omega) \tag{S22}$$

with the normalized ($\int d\omega J_0(\omega) = 1$) function

$$J_0(\omega) = \frac{1}{s_1 + s_2} \sum_{i=1,2} \frac{s_i}{7! 2 \omega_i^4} \omega^3 \mathrm{e}^{-(\omega/\omega_i)^{1/2}}, \tag{S23}$$

Table S3: Atomic partial charges of the ground ($q_k(0)$) and excited ($q_k(1)$) state of Chl *a* in units of the elementary charge e, obtained with (time-dependent) density functional theory using the CAM-B3LYP exchange-correlation functional and a 6-31G(d.p) basis set. The labeling of the heavy atoms is according to the protein data bank file 6M32.

k	$q_k(0)$	$q_k(1)$	k	$q_k(0)$	$q_k(1)$	k	$q_k(0)$	$q_k(1)$
MG	0.981	0.984	O2A	-0.331	-0.331	CBC	-0.223	-0.223
CHA	0.341	0.358	NB	-0.442	-0.437	HBC1	0.06	0.06
CHB	-0.482	-0.467	C1B	0.143	0.115	HBC2	0.06	0.06
HHB	0.153	0.148	C2B	0.26	0.236	HBC3	0.06	0.06
CHC	-0.1	-0.182	C3B	-0.071	-0.11	ND	-0.465	-0.5
HHC	0.107	0.112	C4B	0.094	0.158	C1D	0.09	0.225
CHD	-0.176	-0.341	CMB	-0.55	-0.548	C2D	0.225	0.168
HHD	0.142	0.154	HMB1	0.154	0.153	C3D	-0.286	-0.291
NA	-0.498	-0.529	HMB2	0.154	0.153	C4D	0.073	0.111
C1A	-0.075	-0.054	HMB3	0.154	0.153	CMD	-0.49	-0.48
C2A	0.138	0.146	CAB	-0.106	-0.098	HMD1	0.145	0.145
H2A	0.002	0.003	HAB	0.126	0.125	HMD2	0.145	0.145
C3A	0.048	0.047	CBB	-0.36	-0.375	HMD3	0.145	0.145
H3A	0.03	0.03	HBB1	0.161	0.162	CAD	0.734	0.731
C4A	0.448	0.471	HBB2	0.161	0.162	OBD	-0.483	-0.49
CMA	-0.421	-0.413	NC	-0.342	-0.424	CBD	-0.87	-0.917
HMA1	0.116	0.114	C1C	-0.081	-0.005	HBD1	0.235	0.245
HMA2	0.116	0.114	C2C	0.315	0.329	CGD	0.832	0.838
HMA3	0.116	0.114	C3C	-0.216	-0.231	O1D	-0.527	-0.527
CAA	0.128	0.127	C4C	0.115	0.279	O2D	-0.284	-0.28
HAA1	-0.024	-0.024	CMC	-0.611	-0.609	CED	-0.212	-0.215
HAA2	-0.024	-0.024	HMC1	0.167	0.167	HED1	0.126	0.127
CBA	-0.25	-0.247	HMC2	0.167	0.167	HED2	0.126	0.127
HBA1	0.03	0.028	HMC3	0.167	0.167	HED3	0.126	0.127
HBA2	0.03	0.028	CAC	0.133	0.135	C1	-0.191	-0.191
CGA	0.783	0.783	HAC1	0.005	0.004	H1	0.182	0.182
O1A	-0.544	-0.544	HAC2	0.005	0.004	H2	0.181	0.181

Table S4: $S_0 \rightarrow S_1$ transition partial charges of Chl *a* in units of the elementary charge *e*, obtained with (time-dependent) density functional theory using the B3LYP exchange-correlation functional and a 6-31G(d,p) basis set in units of the elementary charge e. The labeling of the atoms is according to the protein data bank file 6M32.

k	$q_k(0,1)$	k	$q_k(0,1)$
MG	0.019992	CBB	-0.019596
CHA	-0.124346	NC	-0.001481
CHB	0.04739	C1C	-0.056954
CHC	0.075269	C2C	0.008297
CHD	-0.060959	C3C	-0.015875
NA	-0.033081	C4C	0.048831
C1A	0.131879	CMC	0.004958
C2A	0.001229	CAC	0.000113
C3A	-0.007187	CBC	-0.005391
C4A	-0.071689	ND	-0.099372
CMA	-0.005981	C1D	0.08796
CAA	-0.000753	C2D	0.011745
CBA	0.010669	C3D	-0.018422
CGA	-0.013916	C4D	0.136381
O1A	-0.001661	CMD	0.022493
O2A	0.009124	CAD	0.024646
NB	0.06935	OBD	0.015667
C1B	-0.076496	CBD	0.009579
C2B	-0.019158	CGD	-0.00838
C3B	0.035778	O1D	0.005181
C4B	-0.110129	O2D	0.001785
CMB	-0.014811	CED	0.003524
CAB	-0.016202		

Table S5: $S_0 \rightarrow S_1$ transition partial charges of BChl *a*, obtained with (time-dependent) density functional theory using the B3LYP exchange-correlation functional and a 6-31G(d,p) basis set in units of the elementary charge *e*. The labeling of the atoms is according to the protein data bank file 6M32.

k	$q_k(0,1)$	k	$q_k(0,1)$
MG	0.032744	OBB	-0.027316
CHA	-0.12778	CBB	-0.01756
CHB	0.017336	NC	-0.015507
CHC	0.038141	C1C	-0.061299
CHD	-0.034456	C4C	0.092093
NA	-0.054892	C2C	0.018789
C1A	0.125592	C3C	-0.028031
C4A	-0.023652	CMC	-0.005323
C2A	-0.021191	CAC	-0.001195
C3A	0.004964	CBC	0.007707
CMA	-0.009009	ND	-0.097838
CAA	0.009803	C1D	0.079886
CBA	-0.005437	C4D	0.158537
CGA	0.007	C2D	0.00988
O1A	-0.004408	C3D	-0.033559
O2A	0.000759	CMD	0.029166
NB	0.018127	CAD	0.024035
C1B	-0.058411	OBD	0.015289
C4B	-0.035827	CBD	0.005117
C2B	0.005253	CGD	-0.001909
C3B	-0.041193	O1D	0.007174
CMB	-0.027749	O2D	-0.002606
CAB	0.021914	CED	0.006843

and the Huang-Rhys factor S. The parameters for $J_0(\omega)$ are $s_1 = 0.8$, $s_2 = 0.5$, $\hbar\omega_1 = 0.069\,\mathrm{meV}$, $\hbar\omega_2 = 0.24\,\mathrm{meV}$. The Huang-Rhys factor S can be obtained from the temperature dependence of optical spectra. For the FMO protein, we obtained[17] $S = 0.42$. We will assume the same value for the pigments in the RCC.

S1.4 Linear Optical Spectra

The linear absorption $\mathrm{OD}(\omega)$ is given as

$$\mathrm{OD}(\omega) = \sum_a \langle \mathrm{OD}_a(\omega) \rangle_{\mathrm{dis}}\,, \tag{S24}$$

where $\langle \ldots \rangle_{\mathrm{dis}}$ denotes an average over static disorder in site energies. The linear absorption of domain a is given as

$$\mathrm{OD}_a(\omega) \propto \omega \sum_{M_a} |\boldsymbol{\mu}_{M_a}|^2\, D_{M_a}(\omega) \tag{S25}$$

with the transition dipole moment

$$\boldsymbol{\mu}_{M_a} = \sum_{m_a} c_{m_a}^{(M_a)} \boldsymbol{\mu}_{m_a} \tag{S26}$$

where $\boldsymbol{\mu}_{m_a}$ is the local transition dipole moment of pigment m_a. The lineshape function $D_{M_a}(\omega)$ for the optical excitation of the M_ath exciton state is described in eq S3. Further details can be found in the original Ref.[8].

The linear dichroism (LD) spectrum[26,27] is obtained by replacing the square of the transition dipole moment $|\boldsymbol{\mu}_{M_a}|^2$ in eq S25 with the expression $|\boldsymbol{\mu}_{M_a}|^2 \left(1 - 3\cos^2(\Theta_{M_a})\right)$, where Θ_{M_a} denotes the angle between the transition dipole moment $\boldsymbol{\mu}_{M_a}$ and the membrane normal vector. The linear dichroism spectrum of a domain is then given as

$$\mathrm{LD}_a(\omega) \propto \omega \sum_{M_a} |\boldsymbol{\mu}_{M_a}|^2 \left(1 - 3\cos^2(\Theta_{M_a})\right) D_{M_a}(\omega). \tag{S27}$$

The circular dichroism (CD) spectrum reads

$$\mathrm{CD}(\omega) = \sum_a \langle \mathrm{CD}_a(\omega) \rangle_{\mathrm{dis}} \tag{S28}$$

with the spectrum of domain a

$$\mathrm{CD}_a(\omega) \propto \sum_{M_a} R_{M_a} D_{M_a}(\omega) \tag{S29}$$

where the rotational strength R_{M_a} is given as

$$R_{M_a} = \sum_{m_a, n_a} c_{m_a}^{(M_a)} c_{n_a}^{(M_a)} \boldsymbol{R}_{m_a,n_a} \cdot (\boldsymbol{\mu}_{m_a} \times \boldsymbol{\mu}_{m_a}) \tag{S30}$$

The distance vector $\boldsymbol{R}_{m_a,n_a} = \boldsymbol{R}_{m_a} - \boldsymbol{R}_{n_a}$ connects the centers of pigments m_a and n_a.

Figure S1: Optical spectra calculated for alternative set of site energies. Left: Same as Figure 2 of the main text, but using the site energies from CDC calculations (Table S1). Right: Same as Figure 3 of the main text, but using the site energies from CDC calculations (Table S2).

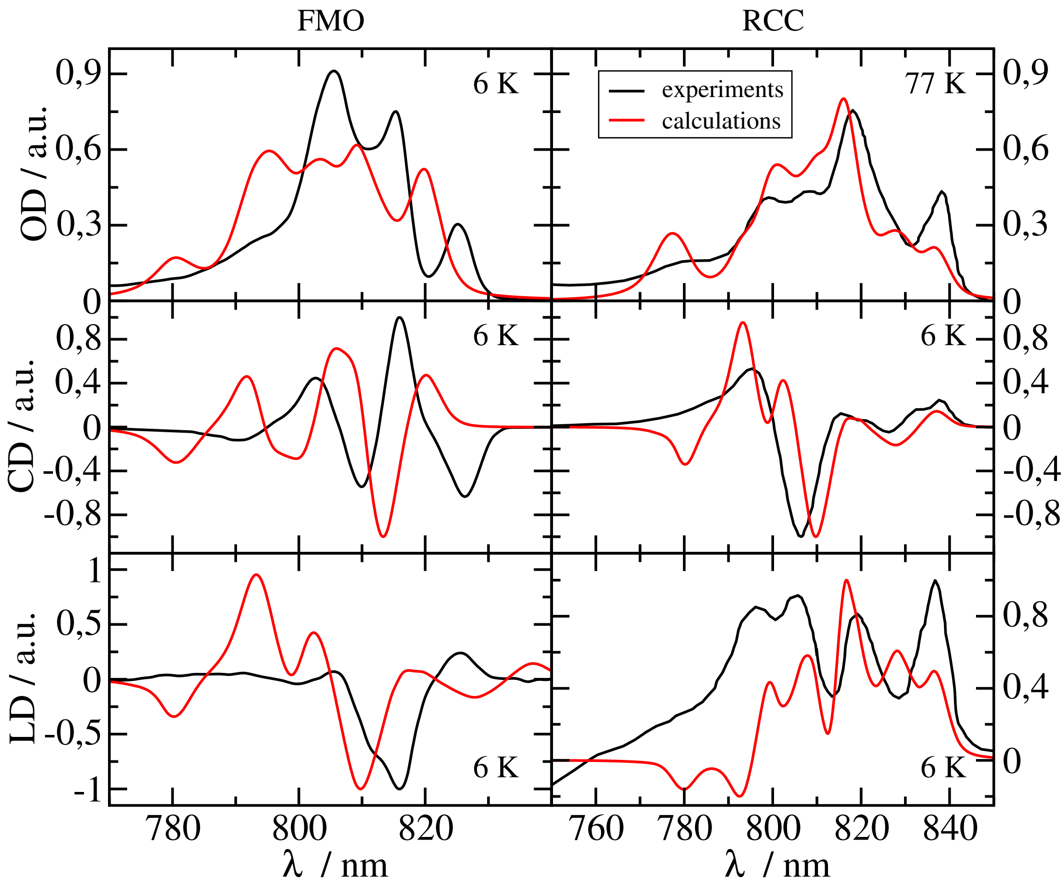

Figure S2: Optical spectra calculated for alternative set of excitonic couplings. Left: Same as Figure 2 of the main text, but for $f^{RF} = 1.00$. Right: Same as Figure 3 of the main text, but using $f^{RF} = 1.00$ in the calculations of excitonic couplings.

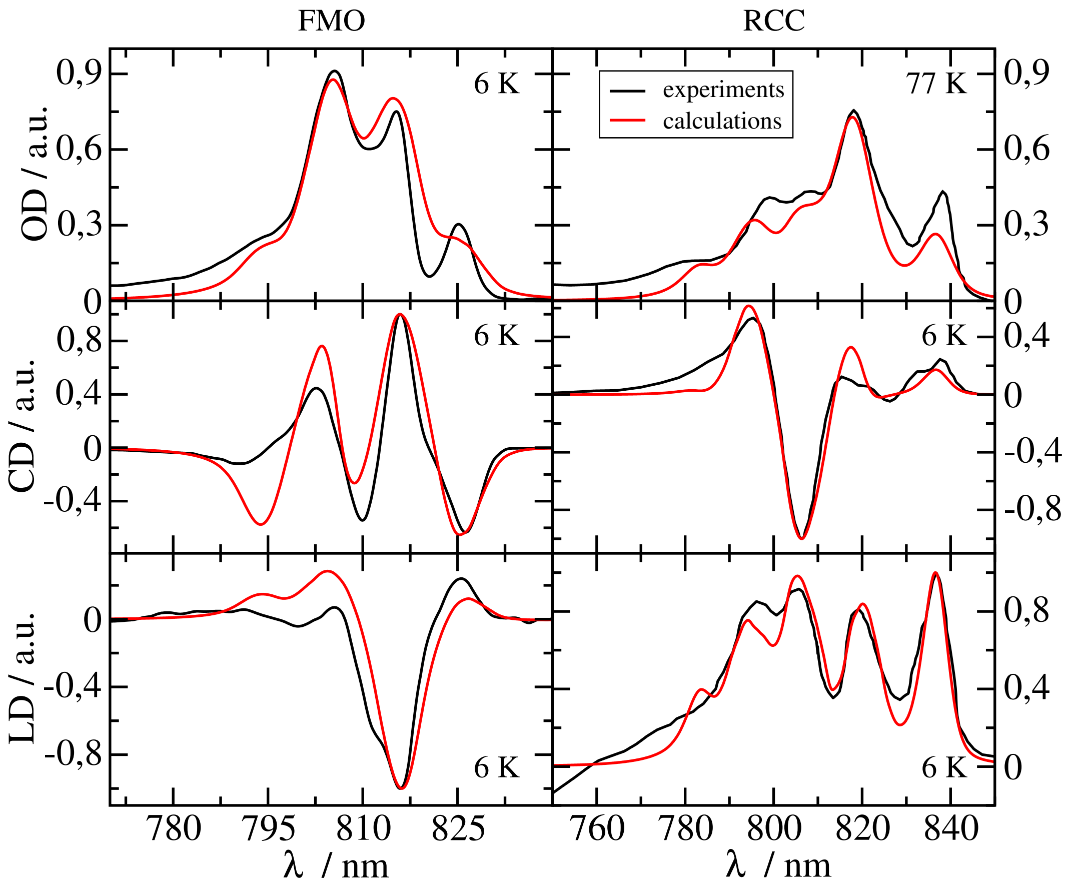

Figure S3: Optical spectra calculated for alternative set of excitonic couplings. Same as Figure S2, but using $f^{\mathrm{RF}} = 1.44$.

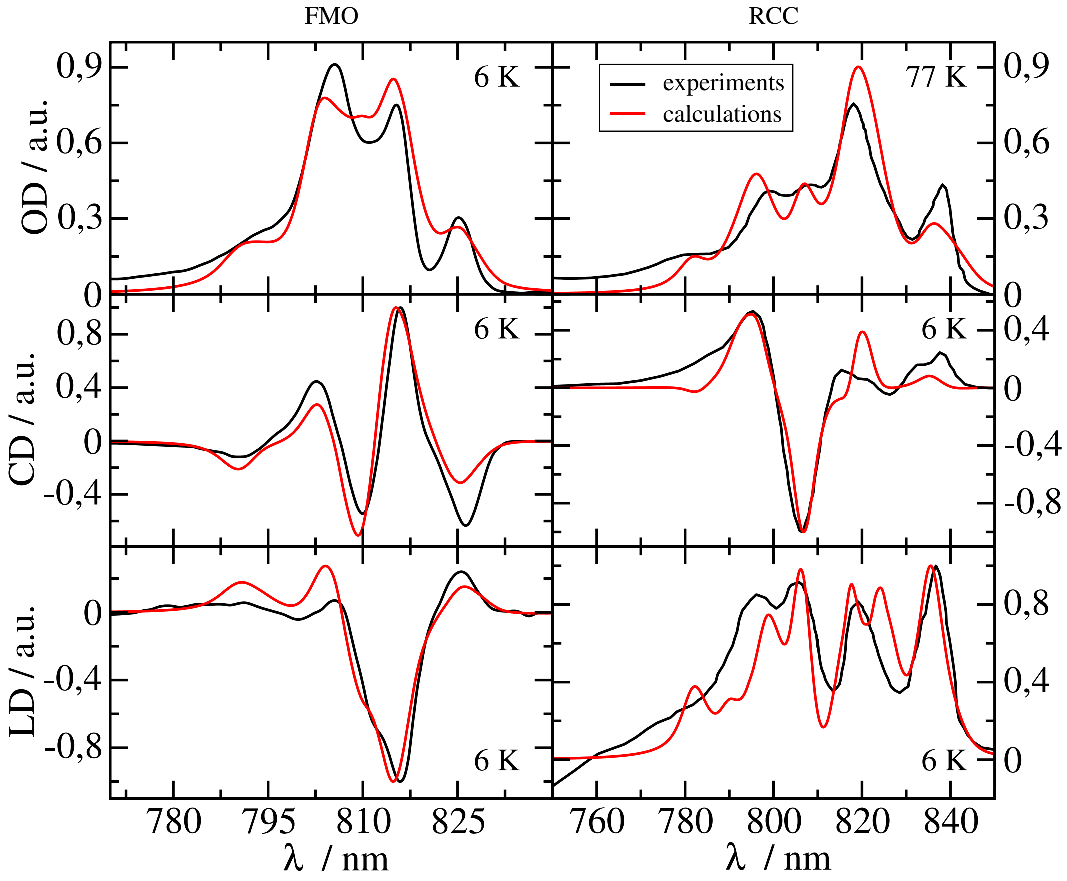

Figure S4: Correlation between different sets of site energies for the FMO protein. Correlation between the site energies of the BChl *a* pigments of the FMO protein obtained from a fit of optical spectra using a genetic algorithm and the site energies calculated with the CDC method based on the cryo-EM structural model[19] (Table S1).

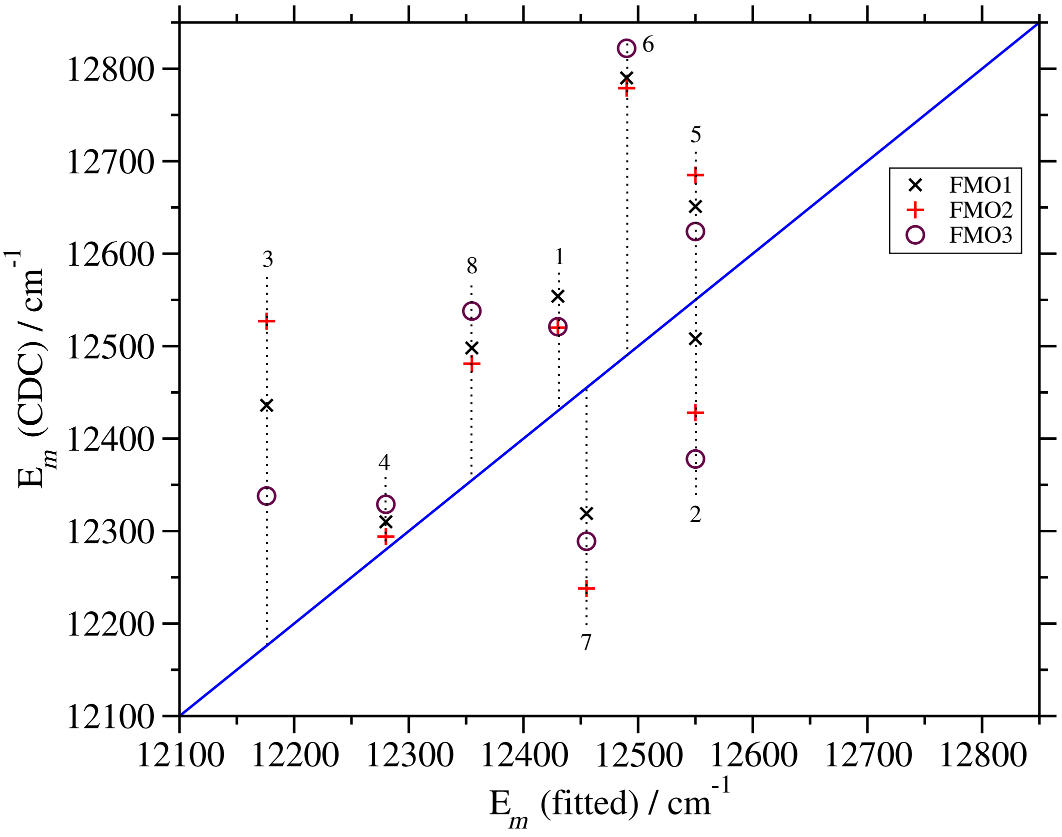

Figure S5: Correlation between different sets of site energies for the RCC. Correlation between the site energies of the BChl a pigments of the RCC obtained from a fit of optical spectra using a genetic algorithm and the site energies calculated with the CDC method based on the cryo-EM structural model[19] (Table S2).

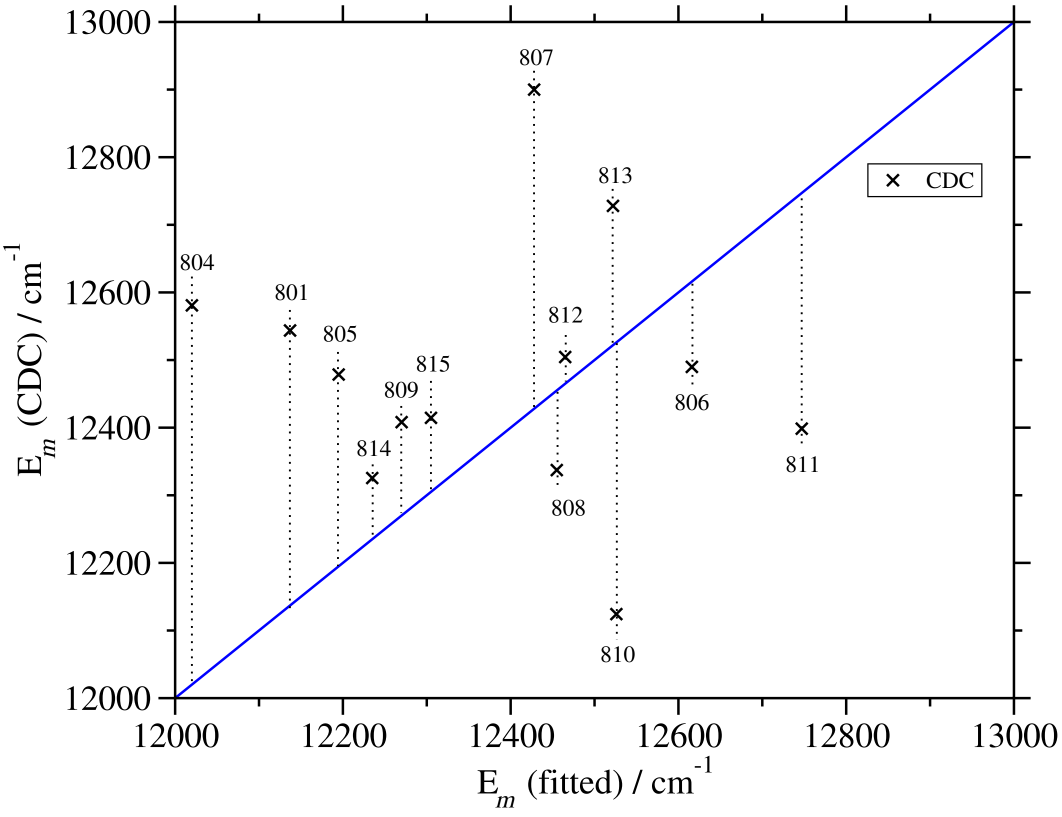

Figure S6: Time constants for inter-exciton domain energy transfer in the FMO-RCC. View on FMO-RCC along the membrane normal. Pigments are colored according to their respective exciton domain. For symmetry reasons, the pigments in RCC-Ant1 and RCC-Ant2 have the same color although they belong to different exciton domains. The colored arrows show the most important excitation energy transfer pathways between different exciton domains with their corresponding transfer times in picoseconds. Graphics prepared using VMD.[28]

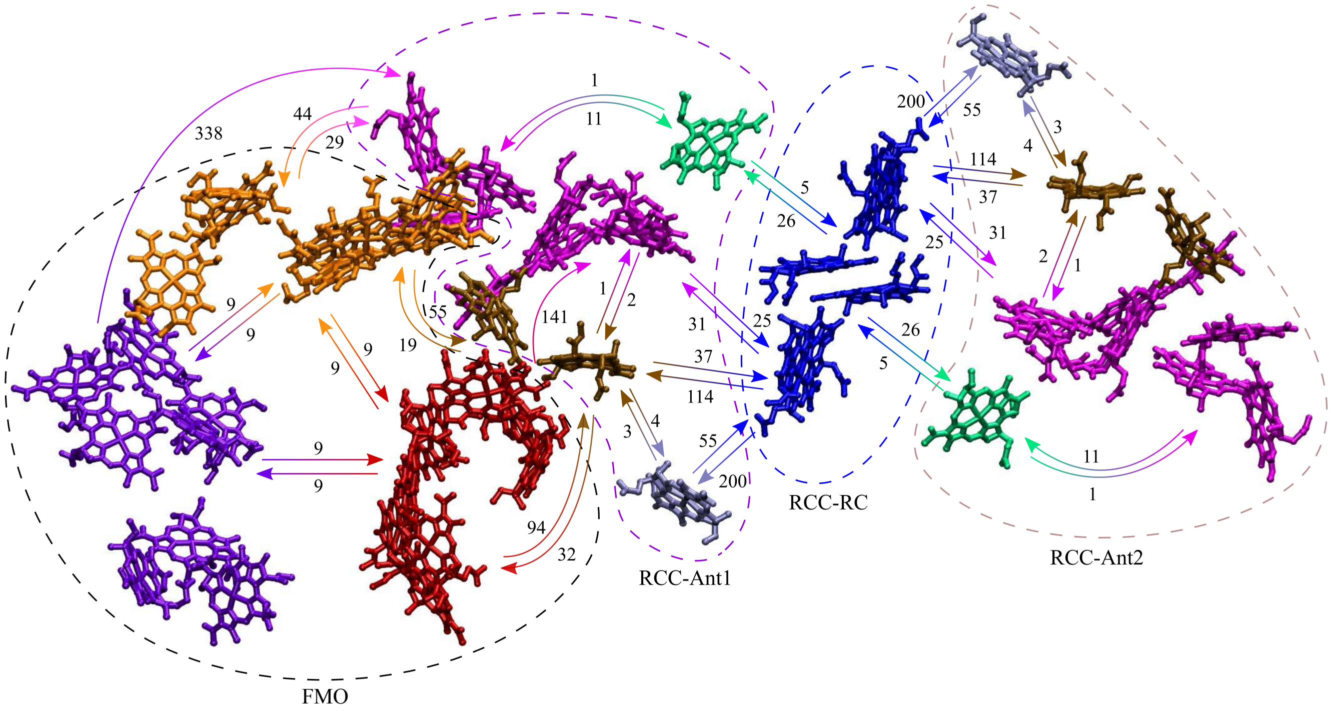

Figure S7: Light harvesting assuming slow electron transfer. Same as Figure 4 of the main text (no quenching), but assuming a primary electron transfer time constant of 25 ps.

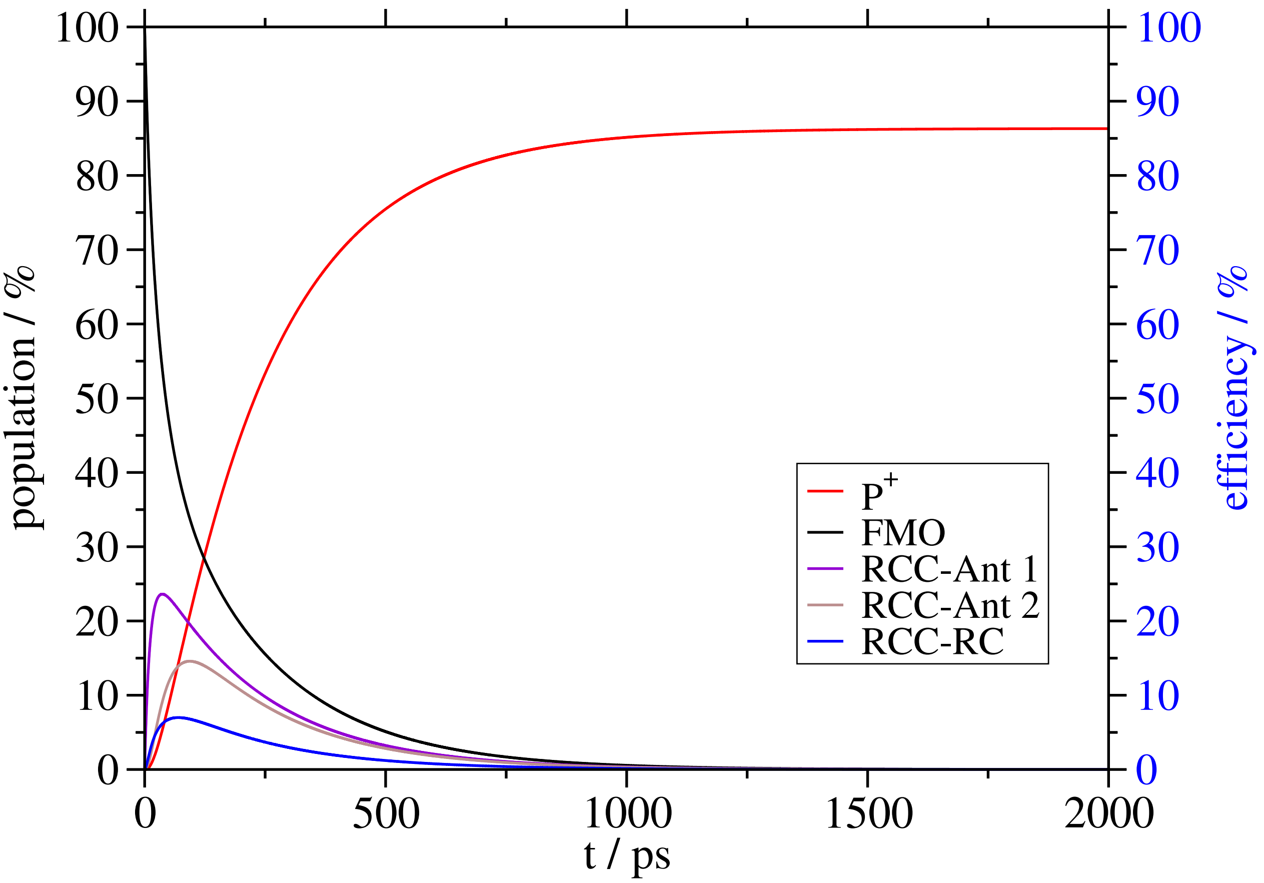

Figure S8: Light harvesting assuming classical nuclear motion. Populations of excited states (black lines) and charge separated state P^+ (red lines) obtained in the original model (same as in Figure 5 of the main text) are compared to those obtained by using a semiclassical Redfield/semiclassical generalized Förster theory ($R_{CL}F_{CL}$) and quantum Redfield/semiclassical generalized Förster theory ($R_{QM}F_{CL}$).

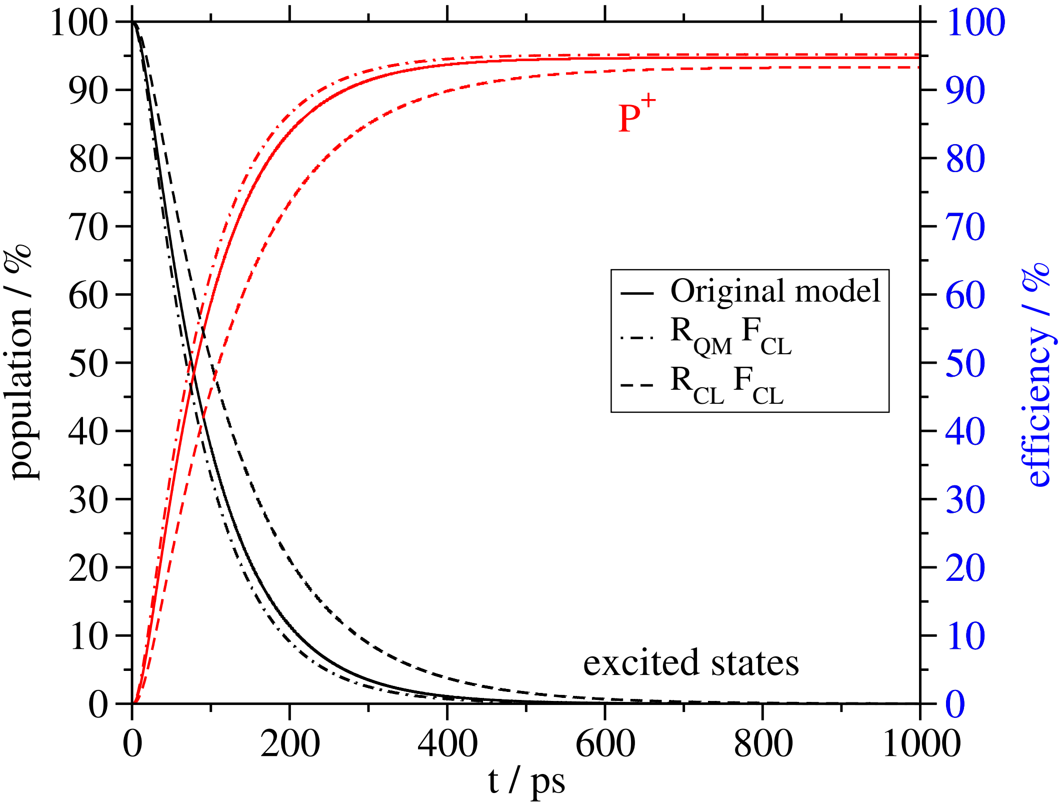

Figure S9: Light harvesting and photoprotection for different sets of excitonic couplings. Left: Population dynamics of the original model in Figure 5 of the main text is compared to those obtained by using different reaction field factors f^{RF} in the calculations of excitonic couplings. Right: Same as left, but under oxidative stress (with quenching).

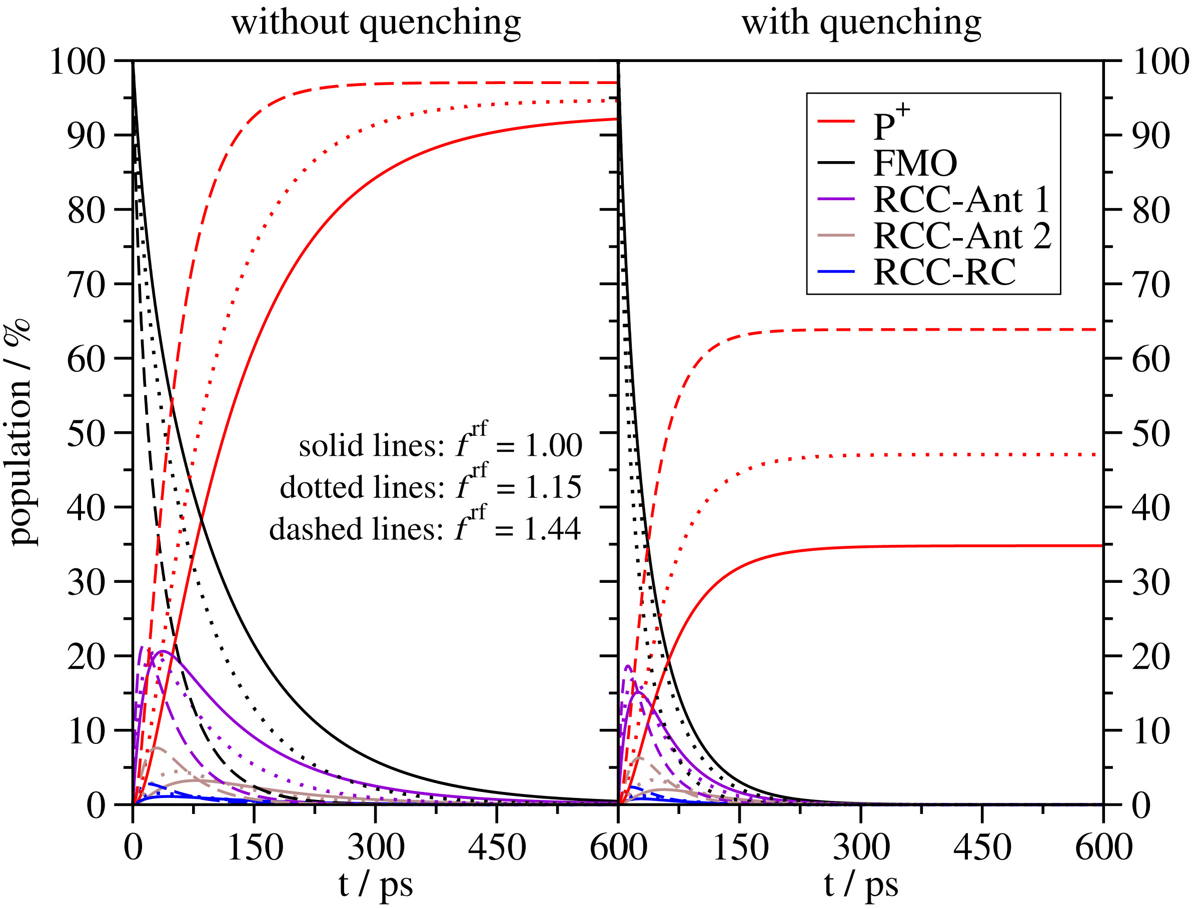

Figure S10: Light harvesting for different sets of site energies. Population dynamics in the FMO-RCC comparing different sets of site energies, excitonic couplings calculated with $f^{\mathrm{RF}} = 1.15$.

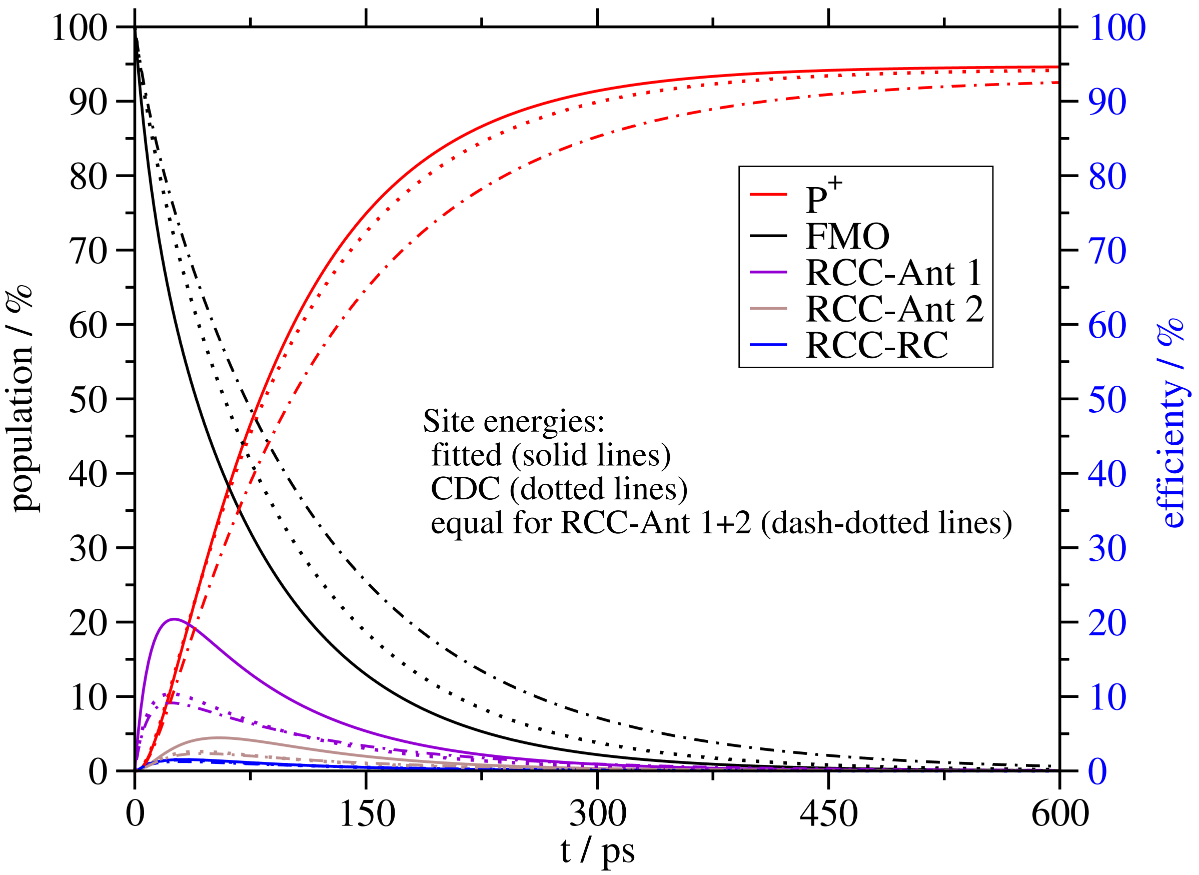

Figure S11: Light harvesting including a second FMO trimer. Same as Figure 4 of the main text (no quenching), but assuming that a hypothetical second FMO protein is bound to the homo-dimeric RCC at a symmetric position with respect to the first. Please note that the populations of FMO1 and FMO2 are practically identical. The same is true for RCC-Ant1 and RCC-Ant2

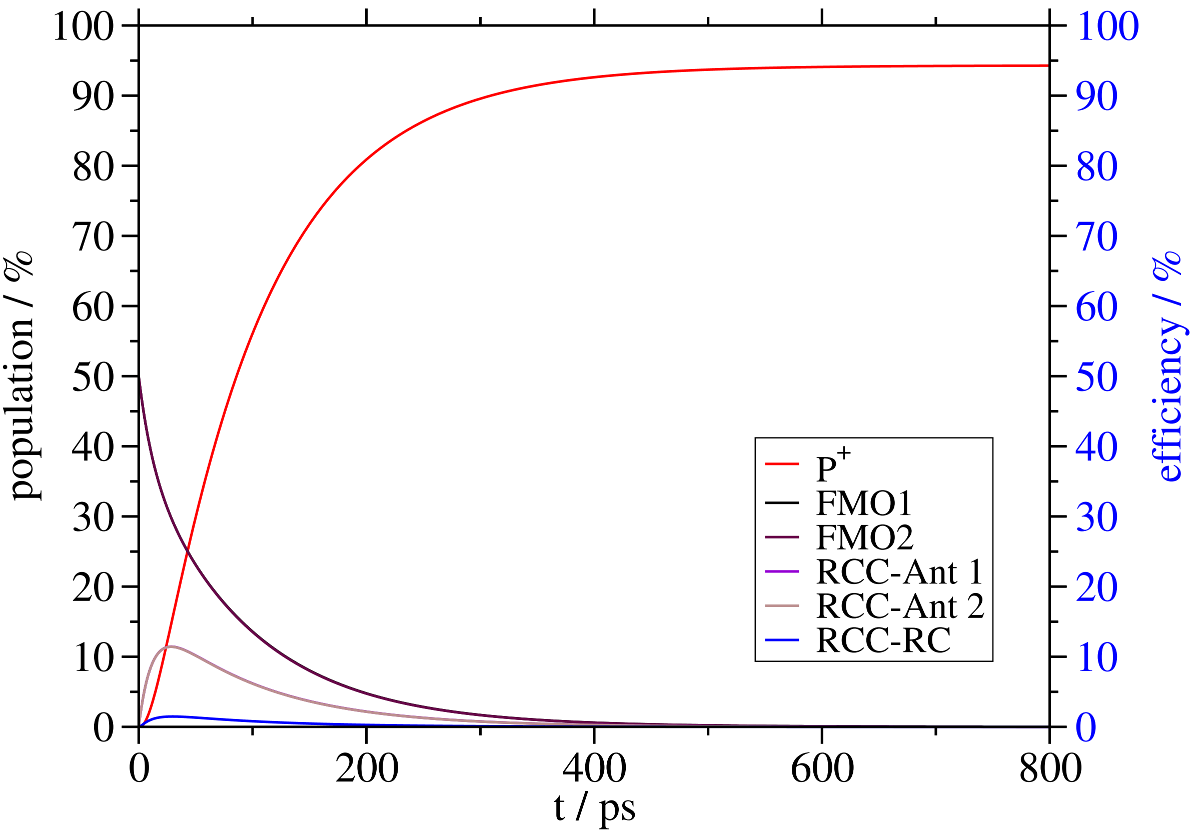

Figure S12: Quenching of excitation energy, isolated FMO protein. Population of excited states in the isolated FMO protein for different quenching scenarios: no quenching (brown line), quenching at BChl 2 only (purple line), quenching at BChl 3 only (black line) and quenching at BChls 2 and 3 (red line). An intrinsic quenching time constant of $\tau_Q = 23$ ps has been inferred from the fluorescence life time as described in section S1.2.

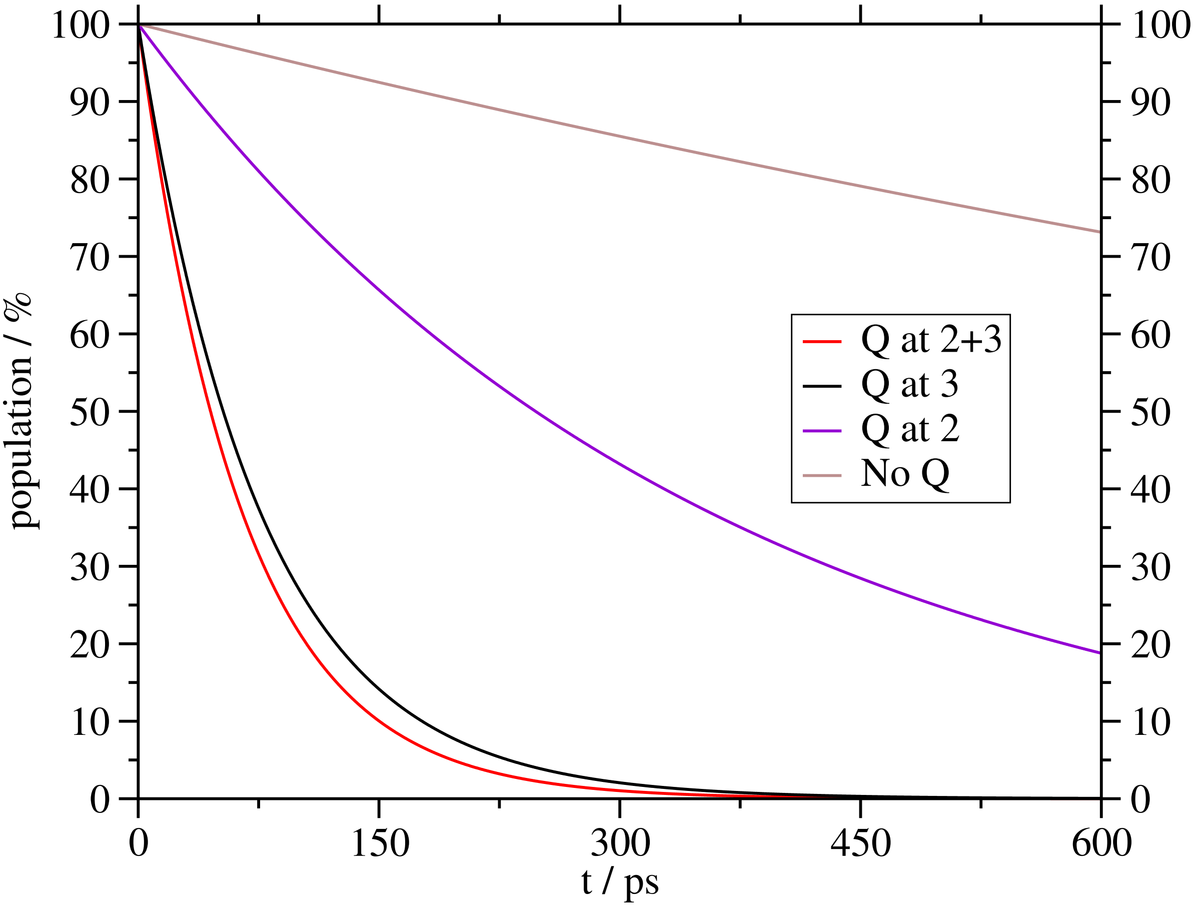

Figure S13: Quenching of excitation energy, FMO-RCC supercomplex. Population of excited states (black lines) and charge separated state P^+ (red lines) in the FMO-RCC supercomplex for different quenching scenarios: no quenching (solid lines), quenching at BChl 2 only (dotted lines), quenching at BChl 3 only (dashed lines) and quenching at BChls 2 and 3 (dash-dotted lines).

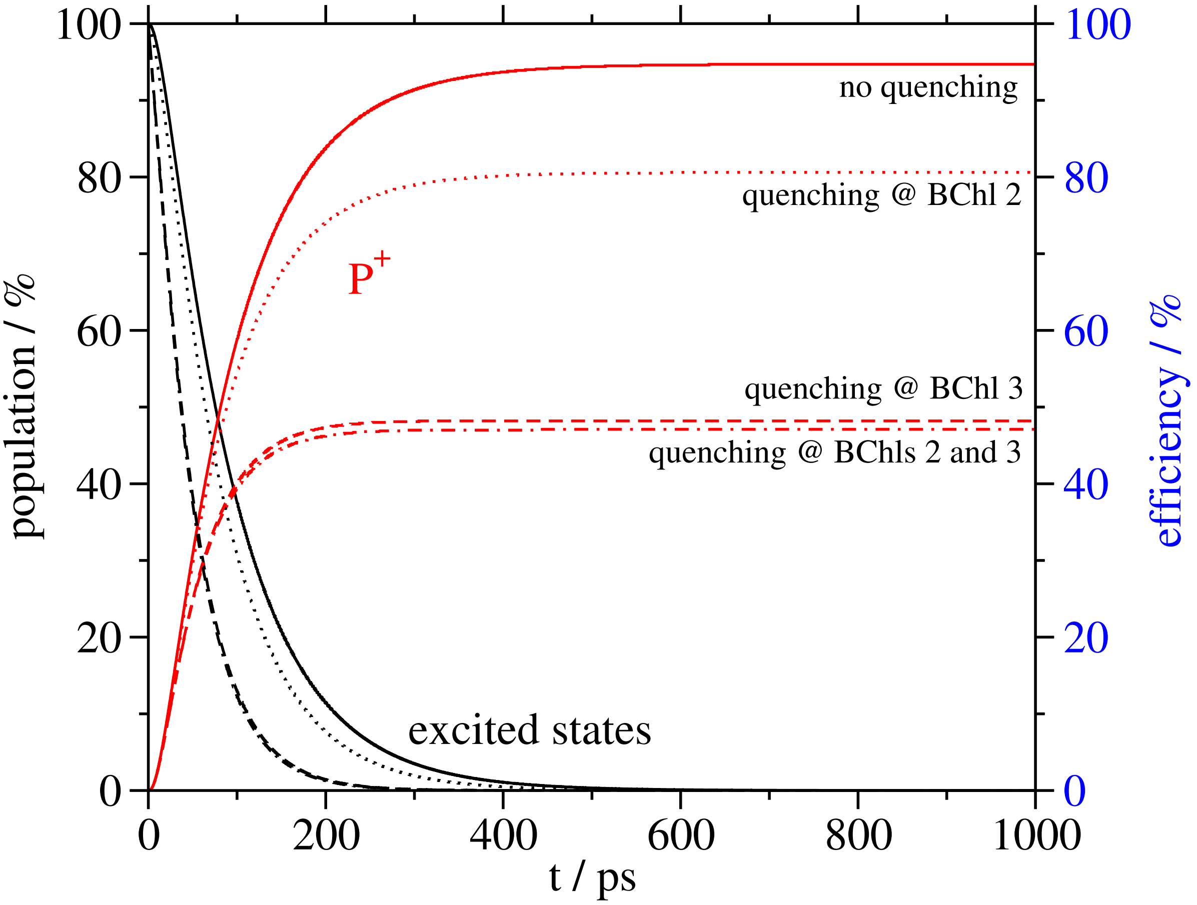

References

[1] T. Renger and F. Müh, *Phys. Chem. Chem. Phys.*, 2013, **15**, 3348–3371.

[2] A. Klinger, D. Lindorfer, F. Müh and T. Renger, *J. Chem. Phys.*, 2020, **153**, 215103.

[3] Z. Fetisova, A. Freiberg, K. Mauring, V. Novoderezhkin, A. Taisova and K. Timpmann, *Biophys. J.*, 1996, **71**, 995–1010.

[4] K. Mukai, S. Abe and H. Sumi, *J. Phys. Chem. B*, 1999, **103**, 6096–6102.

[5] G. D. Scholes and G. R. Fleming, *J. Phys. Chem. B*, 2000, **104**, 1854–1868.

[6] S. Jang, M. D. Newton and R. J. Silbey, *Phys. Rev. Lett.*, 2004, **92**, 218301.

[7] G. Raszewski and T. Renger, *J. Am. Chem. Soc.*, 2008, **130**, 4431–4446.

[8] T. Renger and R. A. Marcus, *J. Chem. Phys.*, 2002, **116**, 9997–10019.

[9] J. Cao, R. J. Cogdell, D. F. Coker, H.-G. Duan, J. Hauer, U. Kleinekathöfer, T. L. C. Jansen, T. Mancal, R. J. D. Miller, J. P. Ogilvie, V. I. Prokhorenko, T. Renger, H.-S. Tan, R. Tempelaar, M. Thorwart, E. Thyrhaug, S. Westenhoff and D. Zigmantas, *Sci. Adv.*, 2020, **6**, eaaz4888.

[10] D. Lindorfer and T. Renger, *J. Phys. Chem. B*, 2018, **122**, 2747–2756.

[11] G. S. Orf, R. G. Saer, D. M. Niedzwiedzki, H. Zhang, C. L. McIntosh, J. W. Schultz, L. M. Mirica and R. E. Blankenship, *Proc. Natl. Acad. Sci.*, 2016, **113**, E4486–E4493.

[12] D. Bashford, *Scientific Computing in Object-Oriented Parallel Environments*, Springer, 1997.

[13] C. Friedl, D. G. Fedorov and T. Renger, *Phys. Chem. Chem. Phys.*, 2022, **24**, 5014–5038.

[14] J. Adolphs, F. Müh, M. E. Madjet and T. Renger, *Photosynth. Res.*, 2008, **95**, 197–209.

[15] D. E. Tronrud, J. Wen, L. Gay and R. E. Blankenship, *Photosynth. Res.*, 2009, **100**, 79–87.

[16] M. Schmidt am Busch, F. Müh, M. E. Madjet and T. Renger, *J. Phys. Chem. Lett.*, 2011, **2**, 93–98.

[17] J. Adolphs and T. Renger, *Biophys. J.*, 2006, **91**, 2778–2797.

[18] S. I. E. Vulto, M. A. de Baat, R. J. W. Louwe, H. P. Permentier, T. Neef, M. Miller, H. van Amerongen and T. J. Aartsma, *J. Phys. Chem. B*, 1998, **102**, 9577–9582.

[19] J.-H. Chen, H. Wu, C. Xu, X.-C. Liu, Z. Huang, S. Chang, W. Wang, G. Han, T. Kuang, J.-R. Shen and X. Zhang, *Science*, 2020, **370**, eabb6350.

[20] Y. Shao, Z. Gan, E. Epifanovsky, A. T. Gilbert, M. Wormit, J. Kussmann, A. W. Lange, A. Behn, J. Deng, X. Feng, D. Ghosh, M. Goldey, P. R. Horn, L. D. Jacobson, I. Kaliman, R. Z. Khaliullin, T. Kuś, A. Landau, J. Liu, E. I. Proynov, Y. M. Rhee, R. M. Richard, M. A. Rohrdanz, R. P. Steele, E. J. Sundstrom, H. L. W. III, P. M. Zimmerman, D. Zuev, B. Albrecht, E. Alguire, B. Austin, G. J. O. Beran, Y. A. Bernard, E. Berquist, K. Brandhorst, K. B. Bravaya, S. T. Brown, D. Casanova, C.-M. Chang, Y. Chen, S. H. Chien, K. D. Closser, D. L. Crittenden, M. Diedenhofen, R. A. D. Jr., H. Do, A. D. Dutoi, R. G. Edgar, S. Fatehi, L. Fusti-Molnar, A. Ghysels, A. Golubeva-Zadorozhnaya, J. Gomes, M. W. Hanson-Heine, P. H. Harbach, A. W. Hauser, E. G. Hohenstein, Z. C. Holden, T.-C. Jagau, H. Ji, B. Kaduk, K. Khistyaev, J. Kim, J. Kim, R. A. King, P. Klunzinger, D. Kosenkov, T. Kowalczyk, C. M. Krauter, K. U. Lao, A. D. Laurent, K. V. Lawler, S. V. Levchenko, C. Y. Lin, F. Liu, E. Livshits, R. C. Lochan, A. Luenser, P. Manohar, S. F. Manzer, S.-P. Mao,

N. Mardirossian, A. V. Marenich, S. A. Maurer, N. J. Mayhall, E. Neuscamman, C. M. Oana, R. Olivares-Amaya, D. P. O'Neill, J. A. Parkhill, T. M. Perrine, R. Peverati, A. Prociuk, D. R. Rehn, E. Rosta, N. J. Russ, S. M. Sharada, S. Sharma, D. W. Small, A. Sodt, T. Stein, D. Stück, Y.-C. Su, A. J. Thom, T. Tsuchimochi, V. Vanovschi, L. Vogt, O. Vydrov, T. Wang, M. A. Watson, J. Wenzel, A. White, C. F. Williams, J. Yang, S. Yeganeh, S. R. Yost, Z.-Q. You, I. Y. Zhang, X. Zhang, Y. Zhao, B. R. Brooks, G. K. Chan, D. M. Chipman, C. J. Cramer, W. A. G. III, M. S. Gordon, W. J. Hehre, A. Klamt, H. F. S. III, M. W. Schmidt, C. D. Sherrill, D. G. Truhlar, A. Warshel, X. Xu, A. Aspuru-Guzik, R. Baer, A. T. Bell, N. A. Besley, J.-D. Chai, A. Dreuw, B. D. Dunietz, T. R. Furlani, S. R. Gwaltney, C.-P. Hsu, Y. Jung, J. Kong, D. S. Lambrecht, W. Liang, C. Ochsenfeld, V. A. Rassolov, L. V. Slipchenko, J. E. Subotnik, T. V. Voorhis, J. M. Herbert, A. I. Krylov, P. M. Gill and M. Head-Gordon, *Mol. Phys.*, 2015, **113**, 184–215.

[21] E. Sigfridsson and U. Ryde, *J. Comput. Chem.*, 1998, **19**, 377–395.

[22] T. Renger, A. Klinger, F. Steinecker, M. Schmidt am Busch, J. Numata and F. Müh, *J. Phys. Chem. B*, 2012, **116**, 14565–14580.

[23] F. Müh, D. Lindorfer, M. Schmidt am Busch and T. Renger, *Phys. Chem. Chem. Phys.*, 2014, **16**, 11848–11863.

[24] T. Renger, I. Trostmann, C. Theiss, M. E. Madjet, M. Richter, H. Paulsen, H. J. Eichler, A. Knorr and G. Renger, *J. Phys. Chem. B*, 2007, **111**, 10487–10501.

[25] T. Renger, M. E. Madjet, A. Knorr and F. Müh, *J. Plant Physiol.*, 2011, **168**, 1497 – 1509.

[26] H. van Amerongen, H. Vasmel and R. van Grondelle, *Biophys. J.*, 1988, **54**, 65–76.

[27] H. van Amerongen, L. Valkunas and R. van Grondelle, *Photosynthetic excitons*, World Scientific Publishing Co. Pte. Ltd., Oxford, UK, 2000.

[28] W. Humphrey, A. Dalke and K. Schulten, *J. Mol. Graph.*, 1996, **14**, 33–38.
